# Water Is and Is Not H_2_O, Depending on Who You Ask: Conceptualizations of Water Vary Across Chemists and Laypeople

**DOI:** 10.1111/cogs.70094

**Published:** 2025-08-05

**Authors:** Claudia Mazzuca, Marta Arcovito, Ilenia Falcinelli, Chiara Fini, Anna M. Borghi

**Affiliations:** ^1^ Department of Dynamic and Clinical Psychology, and Health Studies Sapienza University of Rome; ^2^ Department of Ancient and Modern Civilisations University of Messina; ^3^ Institute of Cognitive Sciences and Technologies Italian National Research Council

**Keywords:** Concepts, Expertise, Psychological essentialism, Abstractness, Natural kinds, Chemistry, Water

## Abstract

Conceptual representations can be shaped by multiple factors, including expertise. In this study, we tested whether the concept of water is represented differently across laypeople and chemists, focusing on psychological essentialism. Essentialized categories are thought to be determined by internal factors (e.g., chemical composition). Previous research suggests laypeople do not essentialize “water.” Here, we sought to verify whether extensive experience with chemicals might lead to more essentialist conceptions. In the first two experiments, participants provided H_2_O estimates, typicality, centrality, and frequency ratings for water examples, which showed that chemists partially incorporate H_2_O in their conceptual representation of “water.” Experiment 3 underlined qualitative differences in the semantic organization of “water” across the two groups using similarity ratings. Experiment 4 consolidated these results with a sentence acceptability task, underlying the importance of chemical composition in determining what counts as “water” for chemists. Finally, Experiment 5 showed that laypeople consider both “H_2_O” and “water” as more abstract compared to chemists. Our results provide evidence on the variability of both psychological essentialism and conceptual representation overall, which can vary as a function of expertise.

## 1 Introduction

There are striking differences in the organization of semantic knowledge across individuals. This variability stems from multiple factors that differentially shape conceptual representations. For instance, recent cross‐sectional research highlights both qualitative and quantitative differences in semantic structure across the lifespan (Wulff, De Deyne, Jones, & Mata, 2022, 2019; Krethlow, Fargier, & Laganaro, 2020; Cosgrove, Kenett, Beaty, & Diaz, 2021), underlining the impact of environmental aspects and cumulative experiences on lexical‐semantic representations. Similar findings emerge from classic cross‐cultural and cross‐linguistic studies, showing both coarse and fine‐grained differences in patterns of categorization across cultural and linguistic groups (Dougherty, 1981; Rosch & Mervis, 1975; Atran & Medin, 2008, for a review, see Kemmerer, 2019). Importantly, differences in the structure of semantic knowledge have also been linked to varying patterns in cognitive performance (Wulff, De Deyne, Aeschbach, & Mata, 2022).

Expertise can be considered a critical driver of conceptual variation. Over the last decades, evidence on the role of expertise—that is, the degree of skill or knowledge within a given domain (Salas, Rosen, & DiazGranados, 2010)—in the consolidation of refined cognitive skills has accumulated (Sala & Gobet, 2017; Medin, Ross, Atran, Burnett, & Blok, 2002; Klein & Hoffman, 2020; Ericsson, 2006; Medin, Lynch, Coley, & Atran, 1997). An exemplary instance pertains to the field of music, wherein it has been documented that proficient musicians exhibit adeptness in recognizing relative pitch (i.e., the relationships between tones, Levitin & Rogers 2005), envisioning musical compositions from notations (Brodsky, Henik, Rubinstein, & Zorman, 2003), demonstrating more consistent recollection of musical pieces in comparison to amateurs (Halpern & Bower, 1982), and displaying quicker abilities in perceiving similarities between musical instruments and tones when compared with novices (Bensafi et al., 2017; Bensafi, Tillmann, Poncelet, Przybylski, & Rouby, 2013). Furthermore, studies on experienced musicians showed that a prolonged experience with certain entities can also modify their neural representation (Hoenig et al., 2011). Not only can expertise boost cognitive performances related to specific tasks, but research suggests that it might also carve up semantic organization. For example, when asked to describe pictures of composite visual scenes related to computers, computer experts produce descriptions involving more salient details about electronic equipment compared to novices (Humphrey & Underwood, 2011). Likewise, in feature listing tasks, bird and dog experts list more specific features for animals in their domain of expertise (Tanaka & Taylor, 1991), suggesting experts may have more detailed conceptual representations of specific items. In a study by Wing, Burles, Ryan, and Gilboa (2022), expert birdwatchers and laypeople were compared in their similarity judgments for various bird species using a sorting task. The researchers found differences in the strategies employed by the two groups to organize their conceptual knowledge within this domain. While laypeople mainly based their similarity judgments on perceptual features of birds like color, experts’ judgments were primarily driven by taxonomic features.

Finally, a further source of conceptual variability might be constituted by concepts’ concreteness ∼ abstractness. Conceptual abstractness differs from the process of abstraction: while abstraction is a process allowing people to generalize over different instances and underlying the formation of both abstract and concrete concepts (e.g., “definition” vs. “flower”), as well as general or specific concepts (e.g., “flower” vs. “dhalia”) (Reilly et al., 2024; Mazzuca et al., 2025), abstractness is a semantic property of concepts ranging from concrete to abstract (Borghi, Mazzuca, & Tummolini, 2025). Compared to concrete concepts, abstract concepts do not refer to a single, bounded, and perceptually identifiable referent (Borghi & Binkofski, 2014), but collect under a single label a multifarious set of situations and experiences (Barsalou & Wiemer‐Hastings, 2005; Davis, Altmann, & Yee, 2020; Reilly et al., 2024). For instance, abstract concepts such as *time* and *space* have been found to vary consistently across cultures (Boroditsky, 2018; Majid, Bowerman, Kita, Haun, & Levinson, 2004), showing how different cultural and linguistic experiences can alter the perception of these domains (Borghi & Mazzuca, 2023). Interestingly, concepts can not only vary across cultures in terms of their conceptual boundaries, but they can also be conceptualized as more abstract or concrete depending on specific experiences with their referents (Mazzuca et al., 2024). In other words, abstractness in conceptual systems can be culturally dependent. To illustrate, Majid, Burenhult, Stensmyr, De Valk, and Hansson (2018) found that Jahai speakers—a hunter‐gatherer community of the Malaysian peninsula—had a very abstract and refined vocabulary to describe odors when compared to Dutch participants, who instead mostly referred to the source of the odors. It has been proposed (Majid & Kruspe, 2018) that the conceptual refinement of these populations for odors derives from the centrality of olfaction in their everyday life: indeed, when exposed to a variety of odors in an environment where the dominant senses (e.g., vision) are partially impaired (i.e., the forest), the sense of smell would have been enriched—consequently shaping the conceptual boundaries of odors. Taken together, these studies suggest concepts and categories are flexibly tailored to contextual, experiential, and cultural aspects (Yee & Thompson‐Schill, 2016; Banks et al., 2023). More to the point, they foreshadow the impact of specific prolonged life experiences on conceptual abstractness—therefore challenging its universality as a semantic construct.

### 1.1 Psychological essentialism and its implications for conceptual representations

Apparently, evidence on conceptual flexibility is hardly reconcilable with theories of psychological essentialism (Medin & Ortony, 1989). Under this account, people tend to think entities in the world possess immutable, stable, and essential properties that make them what they are.^1^ Essentialized categories are thought to reflect fundamental distinctions in nature and to have discrete rather than fuzzy categorical boundaries (Neufeld, 2022). This perspective has been commonly invoked to explain folk beliefs about natural kinds like animals, plants, or well‐defined natural substances like gold (but see Gelman, 2013), and it has been proposed to have remarkable consequences on various cognitive phenomena, including concept representation and categorization. Patterns of psychological essentialism—although varying in their extent and content—have been documented in both children and adults, encompassing natural categories like animals and socially constructed categories like race and gender (review in Gelman, 2004). For instance, when told the story of a kangaroo adopted by a group of goats, and asked whether the kangaroo would be a good hopper or a good climber, preschool children consistently responded that the kangaroo would be a good hopper (Gelman & Wellman, 1991; see also Keil, 1989). Evidence on psychological essentialism has accumulated over the years (see Neufeld, 2022; Rhodes, 2020), leading scholars to suggest that it might be a universal principle governing human cognition—a sort of “reasoning heuristic” (Gelman, 2003; Gelman & Hirschfeld, 1999; Rhodes, Leslie, & Tworek, 2012).

While some cross‐cultural investigations bolstered the argument of essentialism as a cognitive universal (e.g., Atran et al., 2001; Waxman, Medin, & Ross, 2007; Sousa, Atran, & Medin, 2002), a growing body of research now suggests essentialist patterns might not be as stable. For instance, psychological essentialism concerning social categories has been found to vary along a multitude of dimensions, including cultural, contextual, and individual variability. For instance, children from a progressive, university area in the United States were less likely to essentialize gender categories compared to US children from a more conservative area (Rhodes & Gelman, 2009), while Israeli and Northern Irish children were found to engage more than their US counterparts in essentialist reasoning about religious–ethnic groups (Diesendruck, Goldfein‐Elbaz, Rhodes, Gelman, & Neumark, 2013; Smyth, Feeney, Eidson, & Coley, 2017). Consistent variation in social essentialism has also been reported in a sample of adults across the United States and China, where overall Chinese participants tended to essentialize social groups more compared to American participants (Xu, Wen, Zuo, & Rhodes, 2023).

Although most cross‐cultural research focused on social essentialism, there is initial evidence indicating cross‐cultural variation for natural kinds, too. Machery et al. (2023) investigated whether psychological essentialism about biological kinds varies as a function of culture and language. In their first experiment, they gave participants from 10 different countries (among which USA, UK, Italy, Germany, Portugal, Brazil, Indonesia, India) two scenarios featuring the transformation of one botanical kind (lemons that become something similar to oranges after an explosion) and the discovery of a new chemical substance (that looks, smells, and tastes exactly like water but has a different chemical structure), and asked participants to give essentialist versus nonessentialist judgments. They found that compared to Americans, Indonesian and Indian participants were more likely to give essentialist judgments in the lemon scenario, whereas Italian and Portuguese participants were less likely to do so. In the water scenario, instead, Indonesian and Indian participants displayed less essentialist responses than Americans. Furthermore, the responses demonstrated variability based on participants’ level of education. Individuals with a higher level of education displayed more essentialist response patterns, notably within the water scenario. Collectively, these findings underscore the significance of cultural, linguistic, sociodemographic, and experiential factors in influencing psychological essentialism, even in relation to more concrete, biological concepts.

### 1.2 The present study: Differences and similarities in the conceptual representation of water across laypeople and chemists

Psychological essentialism has been challenged in cognitive psychology by a seminal paper addressing the conceptual representation of “water” (Malt, 1994). Altogether, in the case of “water,” theories of psychological essentialism would predict that people's intuitions of what counts as water would be causally linked with underlying natural properties of the liquid under scrutiny—that is, its chemical composition.^2^ Across four studies, Malt investigated whether the presence or absence, and the amount of H_2_O, determines which liquids are called “water.” Participants were asked to judge the proportion of H_2_O in liquids typically called “water” and liquids not called “water” (Experiment 1, Part 2); to provide ratings about how typical, central to human lives, and frequently encountered examples of water are (Experiment 2); to judge the similarity of pairs of water examples (Experiment 3); and to judge the acceptability of sentences targeting three different senses of “water” (Experiment 4). Overall, the results of the study suggested that the tendency to conceptualize something as “water” depends on the cognitive salience of a liquid in participants’ everyday experience rather than on its percentage of H_2_O, hence undermining strong essentialist accounts of knowledge. This is consistent with the view that “special human interests and concerns” (Chomsky, 1995, pp. 22–23), like function, source, and location, rather than H_2_O content, determine the extension of the term “water.” Critics of this approach have instead argued that both coffee and mineral water *are* “water”—in the sense of being conceptualized as H_2_O—, but they are just *called* differently for pragmatic‐Gricean reasons (Abbott, 1997). Under this account, the term “water” can also be used for substances with impurities (but see LaPorte, 1998 for a discussion on “impurities”) because it is a vague term, encompassing different precision standards for defining the amount of impurities accepted for something composed of H_2_O to be considered “water.” Specifically, what counts for something to be called water or not is whether there is another name for the substance, that is, the result of human interests (e.g., “coffee”: a beverage different from water) along with the fact that “impurities” should be essential properties that make the liquids distinct kinds—despite having high H_2_O percentages like coffee—, hence important to mark terminologically (Abbott, 1999). Perhaps mitigating this debate, Bloom (2007) instead proposes that categorization does not follow “either‐or” rules because there are simply too many in‐between cases—with water being one of these. Indeed, “water” can be simultaneously represented as a natural kind with a specific essence (i.e., H_2_O) and as an artifact with specific functions, hence qualifying for what Bloom defines as *hybrid concepts*.

Results from Malt suggested that people think of “water” mostly as an artifact, central to human life, and with specific functions. However, as Malt acknowledged herself (Malt, 1994, p. 58) and in line with evidence on expertise, it may be speculated that individuals who engage in everyday activities that require accurate and specific knowledge of chemical compositions—that is, chemists—might conceptualize natural entities such as “water” based on their chemical properties, and, therefore, more in line with essentialist theories of cognition compared to laypeople. So, the first aim of this study is to assess whether there are differences in the conceptual representation of “water” between chemists and laypeople, such that chemists endorse a more essentialist representation of “water” than laypeople. Putting it into Abbott's (1999) terms, it might be that chemists’ standards for classifying substances composed of H_2_O as water are significantly higher than laypeople's, such that the concept of water and the concept of H_2_O may completely overlap. Furthermore, in keeping with recent perspectives on conceptual representation, we sought to explore whether judgments of abstractness ∼ concreteness for “water” and “H_2_O” also vary as a function of expertise. For instance, it might be that compared to chemists, laypeople perceive “H_2_O” as more abstract, and that they also judge it more abstract than “water”—perhaps due to its scientific connotation. This is also relevant for the essentialist claim overall: indeed, if people think “water” and “H_2_O” point to the same referent, there should be no difference in conceptual abstractness between the two concepts across the board.

To tackle these questions, we conducted a preregistered study composed of five experiments comparing Italian chemists and laypeople on different conceptual tasks like H_2_O estimation in various liquids (Experiment 1, Part 2) typicality ratings (Experiment 2), similarity ratings (Experiment 3), sentence acceptability judgments (Experiment 4), and abstractness ∼ concreteness ratings (Experiment 5), all targeting participants’ conceptual representation of “water.” Specific hypotheses formulated for individual experiments are detailed in the relevant sections. Three of the five experiments (Experiments 1, 2, and 4) use the exact same methods as the original study, except for the fact that stimuli are Italian words, elicited by Italian native speakers (see Experiment 1, Parts 1 and 1.1A). In Experiment 3—that is, the only exploratory experiment among those taken from the original paper—we introduced a slight modification in the methods due to the unavailability of materials from the original study. Please note that, unlike the original study, all the experiments were conducted online. Finally, Experiment 5 directly addresses the abstractness ∼ concreteness research question. For all the experiments—except for Experiment 5—the sample size was based on the original study and duplicated, to get comparable data for both Italian laypeople (vs. English speaking) and chemists (vs. laypeople). The preregistration of the experiments is available at https://osf.io/hrvjw. All the materials, raw data, and analyses scripts of the study are available online at the OSF repository https://osf.io/ycqan/. Data from all experiments were analyzed using R (RCore Team, 2019) and Rstudio (version 4.0.3). Detailed information regarding the statistical analyses performed in each experiment and about participants’ demographics can be found in the Supplementary Appendix and in Supplementary Materials, respectively.

## 2 Experiment 1: Beliefs about H_2_O

The first experiment is composed of three parts examining beliefs about “water” and their relation with H_2_O. Experiment 1, Parts 1 and 1.1A ask, respectively, which liquids laypeople consider to be water, as well as which liquids they would not call “water,” but consider similar to water. According to a strict essentialist perspective, if people think water is pure H_2_O, then any liquid containing ingredients other than H_2_O should not be called “water.” These data are complemented by corpus‐search data as well as observational data (see 3.1.2). In addition, the two experiments were also aimed at collecting the set of examples that would later be used as stimuli for the relevant tests on expertise in the following experiment. Experiment 1, Part 2, assesses whether both laypeople and chemists believe H_2_O is the dominant ingredient of water and nonwater examples. Looser essentialist interpretations (Putnam, 1988 in Malt, 1994) would suggest people would be inclined to call “water” any liquid they believe has H_2_O as a dominant ingredient, so that any liquid with less than 50% of H_2_O would be called differently. The final, and loosest, possibility is that people call “water” liquids with larger H_2_O amounts than liquids they do not call “water,” so that estimated H_2_O amounts between the two should not overlap. Malt's results show that although the judged H_2_O percentage for nonwaters is lower than water examples, it is, however, over 50%, with most nonwater examples having H_2_O as the dominant ingredient. Furthermore, there was a substantial overlap in estimated H_2_O across individual items of the two lists. Here, we seek to replicate these results with laypeople and investigate whether chemists’ estimates are more aligned with essentialist interpretations instead, possibly being specifically based on their scientific knowledge of the compounds.

### 2.1 Experiment 1, Part 1: Water and nonwater examples

#### 2.1.1 Participants

Seventeen participants completed the task for the water condition (*M*_age_ = 23.47; *SD*_age_ = 1.28, Age range = 20–26), and 15 participants completed the task for the nonwater condition (*M*_age_ = 24.33; *SD*_age_ = 1.95, Age range = 22–29). Demographic information concerning birth sex, gender identity, education, and academic background of participants for this and the following experiments can be found in Supplementary Materials (please see S1, Tables S1–S15). For the present and all the following experiments, we recruited laypeople via word of mouth, social media, and snowball sampling. All participants were native Italian speakers.

Ethical approval for this and the following experiments was provided by the Ethics Committee of the Department of Dynamic, Clinical Psychology and Health (Prot. n. 0000754) of Sapienza University of Rome. Participants gave their informed consent prior to participation.

#### 2.1.2 Procedure

We presented two separate samples of participants with two different versions of an online game implemented in Qualtrics. In the water condition, participants were asked to imagine they were playing a word game in which the goal was to guess the name of the type of water that their challenger had in mind. They were told that the trick was that the challenger was not thinking of an easy, obvious example. As a tip, they were told that their challenger was NOT thinking of tap water or ocean water. Participants could type their responses in blank boxes and were allowed 2 min of time for answering. In the nonwater condition, participants were told that they had to guess something that was very similar to water, but was not actually called “water.” As a hint, they were told that their challenger was NOT thinking of vodka or lemonade. Participants were allowed 2 min of time to answer. After the main task, we collected information about age, birth sex, gender identity, education level, birth country, and native language of participants.

#### 2.1.3 Results

In the water condition, participants produced a total of 73 single occurrences. In the “nonwater” condition, participants produced a total of 61 single occurrences. Frequency distributions followed Zipf's law (Zipf, 1935) with few exemplars mentioned more frequently and a long tail of singletons—as is typical of free‐listing data. Looking at the frequencies, it seems that our participants replicated the pattern of results reported in Malt's experiment. In fact, among the water examples, the most frequently mentioned are examples related to everyday life (e.g., *sparkling water*, *bottled water*, *river water*, *distilled water*, along with a consistent number of water brands). It is worth noting that, although not actually water, *white spirit* was listed by 23.53% of participants in the “water” condition—as its Italian equivalent is “acquaragia,” a compound noun with *water* as root. Likewise, *hydrogen peroxide* was also produced by more than 10% of participants, possibly due to the same reason (It: “acqua ossigenata”). One participant also mentioned *Acqua di Giò* (a famous perfume). As for nonwater examples, many included clear water cases such as *sea*, *rain*, and *sweat*. Taken together, these data suggest participants’ conceptualization of water departs from a strict essentialist representation. Table 1 presents the top 20 exemplars listed for water and nonwater, along with their frequency and English translation.

**Table 1:** Italian water and nonwater examples as resulting from the free‐listing along with their English translations, percentages of frequency, and raw frequency

Italian water example	English translation	Percentage of production and raw frequency	Italian nonwater example	English translation	Percentage of production and raw frequency
*acqua frizzante*	sparkling water	58.82 (10)	*mare*	sea	40 (6)
*acqua in bottiglia*	bottled water	23.53 (4)	*ghiaccio*	ice	33.33 (5)
*acquaragia*	white spirit	23.53 (4)	*pioggia*	rain	33.33 (5)
*acqua di fiume*	river water	17.65 (3)	*liquido*	liquid	26.67 (4)
*acqua distillata*	distilled water	17.65 (3)	*aranciata*	orange juice	20 (3)
*acqua dolce*	sweet water	17.65 (3)	*fiume*	river	20 (3)
*acqua Ferrarelle*	Ferrarelle water	17.65 (3)	*lacrime*	tears	20 (3)
*acqua marina*	marine water	17.65 (3)	*lago*	lake	20 (3)
*acqua ossigenata*	hydrogen peroxide	17.65 (3)	*sudore*	sweat	20 (3)
*acqua potabile*	potable water	17.65 (3)	*vapore*	steam	20 (3)
*acqua salata*	salt water	17.65 (3)	*aria*	air	13.33 (2)
*acqua santa*	holy water	17.65 (3)	*bevanda*	beverage	13.33 (2)
*acqua sporca*	dirty water	17.65 (3)	*coca cola*	coke	13.33 (2)
*acqua azzurra*	blue water	11.76 (2)	*oceano*	ocean	13.33 (2)
*acqua calda*	hot water	11.76 (2)	*saliva*	saliva	13.33 (2)
*acqua di fonte*	spring water	11.76 (2)	*sete*	thirst	13.33 (2)
*acqua di sorgente*	babbling brook water	11.76 (2)	*Sprite*	Sprite	13.33 (2)
*acqua Lete*	Lete water	11.76 (2)	*succo*	juice	13.33 (2)
*acqua micellare*	micellar water	11.76 (2)	*the*	tea	13.33 (2)
*acqua minerale*	mineral water	11.76 (2)	*tisana*	herbal tea	13.33 (2)

Among the exemplars of water produced by more than 10% of participants (*n* = 29), seven exemplars were highly semantically related to “water” according to cosine values retrieved from the Word‐Embeddings Italian Semantic Spaces database (WEISSs, Marelli, 2017). These were “bottled water” (cosine = 0.68); “river water” (cosine = 0.65); “distilled water” (cosine = 0.69); “potable water” (cosine = 0.43); “salt water” (cosine = 0.63); “mineral water” (cosine = 0.65); “rain water” (cosine = 0.57).

### 2.2 Experiment 1, Part 1A: Ratings of water and nonwater examples

The free‐listing data provided an initial glimpse of which liquids people consider to be water examples, and which liquids instead do not qualify as such. Given the variability of responses, and since participants also generated examples that did not seem to be sincere (see also Malt, 1994), the two lists of water and nonwater examples were presented to a separate set of participants to be rated. Specifically, we aimed for water examples that were considered to be “truly examples of water” by the majority of participants and for nonwater examples that were similar to water in some respects (e.g., potability, odor, color), but that are not—and are not called—“water.”

#### 2.2.1 Participants

Examples of water were rated by 15 participants (*M*_age_ = 22.86; *SD*_age_ = 1.76, Age range = 18–25), as were examples of nonwater (*M*_age_ = 23.80; *SD*_age_ = 2.75, Age range = 19–32).

#### 2.2.2 Procedure

We presented two separate samples of participants with two different versions of an online questionnaire implemented in Qualtrics. In the water condition, participants were asked to judge whether the water examples they were given (*N* = 61) were truly examples of water (“yes”) or not (“no”). In the nonwater condition, participants were asked instead to rate the similarity of each nonwater example (*N* = 53) to “water” (1 = “low similarity”; 7 = “high similarity”). Lists of water and nonwater examples were extracted from the free‐listing task, after removing clearly unrelated items (e.g., *thirst*, *life*). After the main task, we collected information about age, birth sex, gender identity, education level, birth country, and native language of participants.

#### 2.2.3 Results

The results of the free‐listing and the rating task served as the basis for the creation of the list of stimuli that was subsequently used in the following experiments, and were complemented by other sources of information (see Malt, 1994). First, we performed a corpus‐based search for terms co‐occurring with “water” in Italian. To do that, we used Italian semantic databases, that is, Word‐Embeddings Italian Semantic Spaces (WEISSs, Marelli, 2017) and the Italian version of the Small World of Words (https://smallworldofwords.org/en/project/visualize, De Deyne, Navarro, Perfors, Brysbaert, & Storms, 2019) to retrieve a set of examples of water. Words selected from WEISSs were all highly related to water, with a cosine distance from the concept “water” ranging from 0.43 to 0.72. Finally, we also looked for examples in conversations, newspapers, television, and grocery or other store shelves.

From the rating task, we selected from the list of “water” examples only those that received at least 10 “yes,” and that did not have a different lemmatization in Italian (e.g., “sudore,” *sweat*). This resulted in a list of 27 examples. We added to these *n* = 20 examples taken from Malt's original paper, most of which also appeared in the Italian databases of semantic similarity. This resulted in a final list of 47 water examples.

From the list of “nonwater” examples, we selected items that received a mean similarity of 3.00 and that did not name a type of water (Malt, 1994). This resulted in a list of 17 examples. We added to these *n* = 19 examples taken from Malt and *n* = 11 examples from observation, following Malt's procedure. This resulted in a final list of 47 nonwater examples. Among nonwater examples produced by Italian participants in Experiment 1, Part 1 that received high similarity scores to the category of “water” in Experiment 1, Part 1A, two are compound names formed by the Italian root *acqua* (i.e., *acqua ossigenata*, en: “hydrogen peroxide” and *acquaragia*, en: “white spirit”). For the following studies, these instances were substituted with the Italian equivalent of “hydrogen peroxide” (*perossido di idrogeno*, a different Italian term indicating hydrogen peroxide but less commonly used than “acqua ossigenata”) and “eye drops” (it: *collirio*), respectively, which are also present in Malt (1994). For the complete list of water and nonwater examples, please see Supplementary Appendix, Table S1 (Supplementary Material).

### 2.3 Experiment 1, Part 2: Chemists and laypeople judgments of H_2_O

Once we have established the most salient water and nonwater examples for Italian speakers through free‐listing and rating tasks, we can now turn to assess whether there are differences in estimated percentages of H_2_O across laypeople and chemists.

Malt's results indicated that most of the water examples were judged as having 89% or less H_2_O, and at the same time that the majority of nonwater examples were judged to have H_2_O as their dominant ingredient—suggesting that the presence of H_2_O alone is not sufficient for explaining what people believe to be water. In this experiment, we sought to assess whether this is also true for chemists.

We hypothesized that if expertise does not affect the conceptualization of “water,” both laypeople and chemists would judge water examples to have higher H_2_O percentages than nonwaters, but would judge most nonwaters examples to have a percentage of H_2_O > 50%—hence considering H_2_O the dominant ingredient also of nonwater examples (Malt, 1994). By contrast, if expertise affects the conceptualization of “water,” both laypeople and chemists would judge water examples to have higher H_2_O percentages than nonwaters, but chemists would judge most nonwater examples to have a percentage of H_2_O < 50%, or in line with the actual H_2_O percentage of the liquid.

#### 2.3.1 Participants

##### 2.3.1.1 Nonchemists

Forty‐seven participants took part in the experiment. Data from one participant in the nonwater condition were removed because the participant did not report their age. In addition, one participant in the nonwater condition indicated “chemical sciences” as their academic background, and so data from this participant qualified for the chemists group. Water examples were evaluated by 23 participants (*M*_age_ = 42.86; *SD*_age_ = 18.25; Age range = 25–70). Nonwater examples were evaluated by a total of 22 participants (*M*_age_ = 35.27; *SD*_age_ = 13.99, Age range = 23–68).

##### 2.3.1.2 Chemists

Forty‐five participants took part in the experiment. Throughout the whole research, chemists were recruited targeting Italian Chemistry Departments by directly emailing faculty members and through Chemistry national scientific associations like SCI (Italian Chemistry Association) and its ECRs section (SCI Giovani). Information about the frequency with which chemists interact with chemicals for the present and the following studies can be found in S2 (Table S16).

Water examples were evaluated by 23 participants (*M*_age_ = 39.47; *SD*_age_ = 14.25; Age range = 24–66). Nonwater examples were evaluated by a total of 23 participants (*M*_age_ = 37.43; *SD*_age_ = 12.02, Age range = 24–63).

#### 2.3.2 Procedure

Participants were presented with an online questionnaire implemented in Qualtrics, composed of three sections. In the first section, participants were first asked to estimate the percentage of H_2_O of either water or nonwater examples (between participants). The order of the exemplars was randomized across participants. Then, in the second section, we collected abstractness ∼ concreteness ratings from both samples for the concepts “H_2_O” and “water.” The last section of the questionnaire contained sociodemographic questions (age, birth sex, gender identity, level of education) as well as a question on academic background. The academic background was assessed using Italian scientific areas categories (*N* = 14). Participants who indicated their academic background was in chemical sciences were also presented with a question asking how many times a week they handle or interact with chemical substances or compounds (never = less than once a week; rarely = between once and twice a week; often = from two to three times a week; very often = almost every day; always = every day). Finally, we collected information related to chemophobia and knowledge of toxicological principles using Italian versions of validated questionnaires (Bearth, Kwon, & Siegrist, 2021; Bearth, Saleh, & Siegrist, 2019). The Italian version of the questionnaires is available in the Supplementary Materials (please see S3 Tables S17 and S18) and in the OSF repository. Analyses and descriptive statistics from the questionnaires are also reported in the Supplementary Material (S7).

#### 2.3.3 Results

Before turning to the analyses of interest, we report descriptive statistics of the estimates provided by the two groups for each type of liquid.

##### 2.3.3.1 Water examples

Laypeople's H_2_O estimated percentages of water examples ranged from 46.13 to 82.43, with an average estimate of 68.69 (*SD* = 25.24). Chemists’ H_2_O estimated percentages of water examples were overall higher and more coherent, ranging from 84.65 to 99.52, with an average estimate of 96.08 (*SD* = 5.48).

##### 2.3.3.2 Nonwater examples

**Fig. 1:**
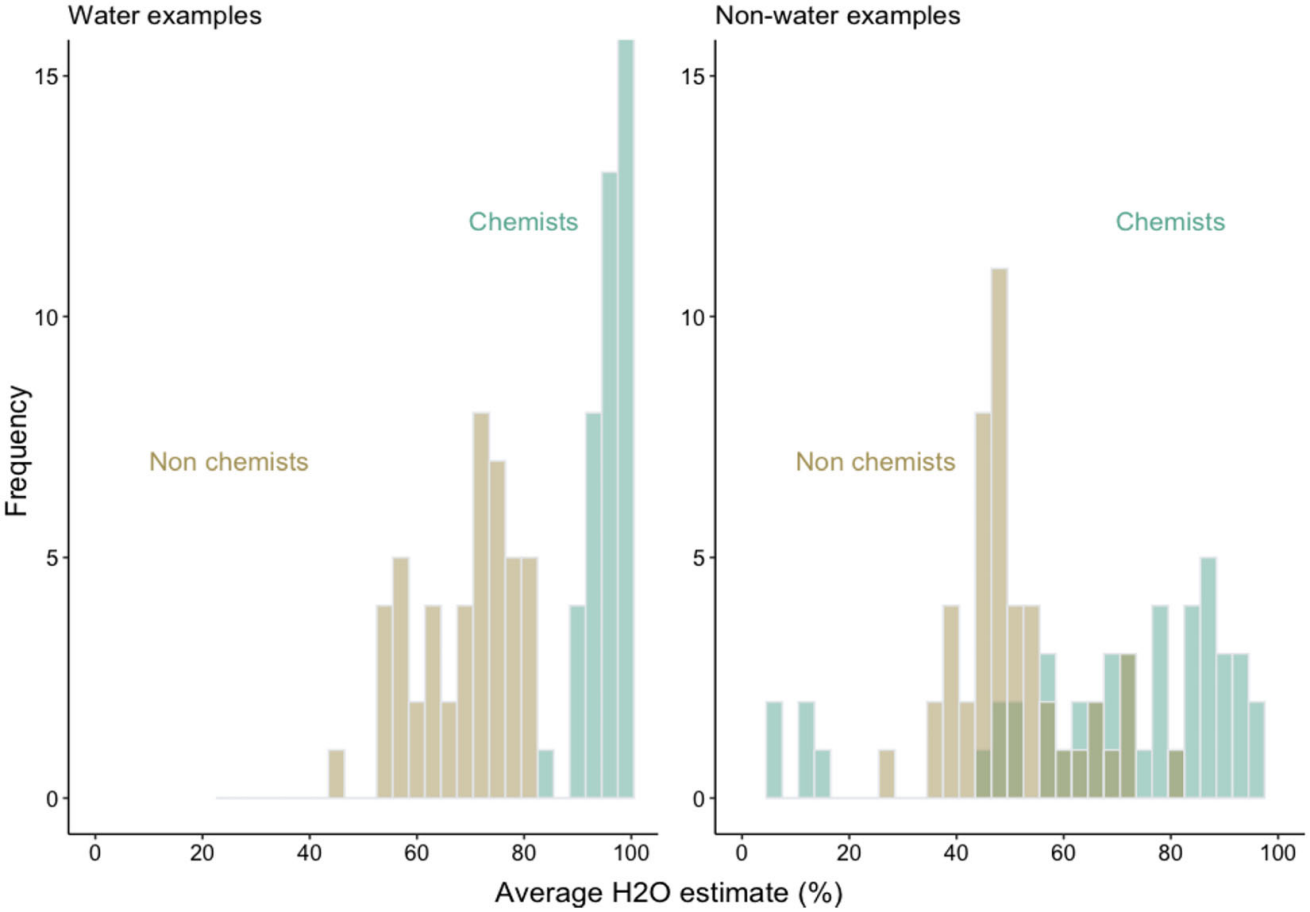
Distribution of estimated H_2_O percentages of water and nonwater examples provided by nonchemists and chemists.

Laypeople's H_2_O estimated percentages of nonwater examples ranged from 26.41 to 81.86, with an average estimate of 50.60 (*SD* = 26.92). Chemists’ H_2_O estimated percentages of nonwater examples showed a wider distribution, ranging from 6.30 to 96, with an average estimate of 67.79 (*SD* = 20.60) (see Fig. 1).

The model showed a main effect of Group, $\chi^{2}$(1) = 53.402, *p* = .015, *b* laypeople = −17.195, 95% CI [−25.72, −8.67], confirming that overall chemists provided higher H_2_O percentage estimates (*M* = 81.93; *SD* = 27.43) compared to nonchemists (*M* = 59.84; *SD* = 29.01). We also found a main effect of Liquid Type, $\chi^{2}$(1) = 34.663, *p* < .0001, *b* water = 28.281, 95% CI [18.53, 38.03], showing that water examples received higher H_2_O percentage estimates (*M* = 82.38; *SD* = 23.85) across groups, compared to nonwater examples (*M* = 59.38; *SD* = 31.77). The two‐way interaction between Group and Liquid Type did not reach significance, $\chi^{2}$(1) = 2.775, *p* = .095, *b* = −10.189, 95% CI [−22.18, 1.80]. So, as expected, water examples were always judged as having higher percentages of H_2_O—replicating Malt's results. However, we did not find the difference in H_2_O estimates provided by the two groups as a function of the type of liquid we predicted.

The second part of our hypothesis specifically relates to the alignment between H_2_O estimates provided by the two groups for nonwater examples and their actual H_2_O percentages. We reasoned that chemists might judge most nonwater examples to have H_2_O percentages lower than 50%, or more in line with their actual H_2_O percentages. To this end, we retrieved H_2_O percentages for each nonwater example using international databases (e.g., https://www.europeanhydrationinstitute.org/nutrition_and_beverages/). Two independent professional chemists double‐checked the list and provided estimates for missing instances, relying where possible on data from patents. On average, we found that nonwater examples contained 75.51% (*SD* = 30.71) of H_2_O. This might explain in part the overall group difference found in the model, showing that chemists, on average, provided higher H_2_O judgments. In fact, 39 out of 48 nonwater examples did indeed contain more than 50% of H_2_O. Laypeople's H_2_O estimates were positively correlated with actual H_2_O percentages of the liquids, *t*(45) = 5.744, *p* = .006, *r* = .65, as were chemists’ estimates, *t*(45) = 13.053, *p* < . 001, *r* = .88—with higher association strength compared to that of laypeople.

#### 2.3.4 Summary

To sum up, this experiment did not provide conclusive evidence on the role of expertise in the conceptualization of “water.” In fact, we found that both chemists and laypeople gave higher H_2_O estimates for water, but there was no difference across chemists and laypeople in H_2_O estimates depending on the liquid type (water vs. nonwater). However, results of the correlations between true estimates of H_2_O percentages and estimates provided by chemists and nonchemists suggest there might be fine‐grained differences—as chemists’ estimates correlated more strongly with true H_2_O estimates than nonchemists’ estimates, and were more consistent with reported percentages. In addition, chemists’ H_2_O estimates for water examples were, on average, higher than 90%, with a very restricted range. On the other hand, laypeople's estimates did not exceed 70%, and the range of H_2_O judgments was broader compared to that of chemists. This might also be an indication that chemists’ conceptualization of water is more centered around H_2_O than that of laypeople.

## 3 Experiment 2: Typicality, centrality, and frequency ratings

The previous experiment showed that the sole presence of H_2_O cannot determine whether a liquid is conceptualized as water or not, regardless of the participants’ expertise. To further probe the conceptual dimensions that mostly explain the belonging of a liquid to the category of “water,” we used typicality, centrality, and frequency ratings. These measures are thought to tap into conceptual dimensions that are relevant for categorical representation (e.g., Rosch & Mervis, 1975). We will compare the results of these tasks to those from Experiment 1, Part 2 to examine whether typicality, centrality, and frequency scores align with percentages of H_2_O estimates in both samples or not.

Malt's results showed that typicality, centrality, and frequency ratings correlate with H_2_O estimates, but typicality ratings correlated more strongly with centrality ratings than with H_2_O estimates, once again suggesting that other factors, rather than chemical structure, underlie the conceptual representation of water. Consistent with that, we hypothesized that if expertise does not affect the conceptualization of “water,” both laypeople and chemists’ ratings of typicality, centrality, and frequency would positively correlate with estimates of H_2_O provided by two different groups of laypeople and chemists. Centrality ratings of both samples would correlate more strongly with typicality ratings compared to estimates of H_2_O (Malt, 1994). By contrast, if expertise affects the conceptualization of “water,” both laypeople and chemists’ ratings of typicality, centrality, and frequency would positively correlate with estimates of H_2_O, but while laypeople's centrality ratings would correlate more strongly with typicality ratings, chemists’ typicality ratings would correlate more strongly with estimates of H_2_O.

### 3.1 Participants

#### 3.1.1 Nonchemists

Typicality ratings were provided by 26 nonchemists (*M*_age_ = 32.15; *SD*_age_ = 13.57; Age range = 23–60), centrality ratings were provided by 20 nonchemists (*M*_age_ = 25.95; *SD*_age_ = 4.50; Age range = 22–38), and frequency ratings were provided by 20 nonchemists (*M*_age_ = 30.2; *SD*_age_ = 11.77; Age range = 20–61).

#### 3.1.2 Chemists

Typicality ratings were provided by 26 chemists (*M*_age_ = 37.46; *SD*_age_ = 11.04; Age range = 24–63), centrality ratings were provided by 20 chemists (*M*_age_ = 39.75; *SD* = 14.96; Age range = 24–81), and frequency ratings were provided by 20 chemists (*M*_age_ = 38.45; *SD*_age_ = 9.89; Age range = 24–64).

### 3.2 Procedure

The questionnaires were composed of three sections. In the first section, participants were asked to rate how typical (1 = low typicality; 7 = high typicality) examples of water are for the category of “water” (Task A), how central (1 = not very central; 7 = very central) they are in human life (Task B), or how frequently (1 = not frequently; 7 = very frequently) they interact with them (Task C). Each participant completed only one Task (between participants), and the order of the examples was randomized across participants. The remaining two sections were identical to those of Experiment 1, Part 2 (see 2.3.2).

### 3.3 Results

Overall, the internal consistency of ratings provided by both groups for typicality, centrality, and frequency was excellent, ranging from *α* = .89 to *α* = .98 (for all the values, please see Supplementary Material—Table S19a).

#### 3.3.1 Nonchemists

Typicality ratings correlated with centrality ratings, *t*(45) = 12.216, *p* = .007, *r* = .87, capturing .76 of variance. By contrast, there was no relation between typicality ratings and H_2_O estimates (i.e., laypeople and chemists of Experiment 1, Part 2, henceforth, H_2_O estimates), *t*(45) = −0.213, *p* = .832, *r* = −.31. Overall, the proportion of variance accounted for by this relation was very low (.001).

Centrality ratings did not correlate with H_2_O estimates, *t*(45) = −0.245, *p* = .807, *r* = .03, with a very low proportion of variance explained (.001).

Adding H_2_O estimates as a further predictor in the model featuring typicality as a dependent variable and centrality as a predictor did not significantly improve the percentage of variance explained by the model with centrality alone, Multiple *R* ^2^ = .77, H_2_O estimates *b* = .01, SE = .01, *t*(44) = 1.176, *p* = .246. Instead, frequency added good predictive power to the model with centrality as a predictor, Multiple *R* ^2^ = .90, frequency *b* = .69, SE = .08, *t*(44) = 8.132, *p* = .0001.

#### 3.3.2 Chemists

Typicality ratings correlated with centrality ratings, *t*(45) = 8.951, *p* = .016, *r* = .80, capturing .65 of variance. Instead, there was no relation between typicality ratings and H_2_O estimates, *t*(45) = 1.672, *p* = .101, *r* = .24. Overall, the proportion of variance accounted for by this relation is .05.

Centrality ratings did not correlate with H_2_O estimates, *t*(45) = 1.954, *p* = .056, *r* = .27, with a very low proportion of variance explained, .069.

Adding H_2_O estimates as a further predictor in the model featuring typicality as dependent variable and centrality as predictor significantly improved the percentage of variance explained by the model with centrality alone, Multiple *R* ^2^ = .67, H_2_O estimates *b* = .05, SE = .02, *t*(44) = 2.054, *p* = .045, and so did frequency, Multiple *R* ^2^ = .69, frequency *b* = .20, SE = .08, *t*(44) = 2.394, *p* = .021. These and all the other correlations, along with their confidence intervals, are reported in S4, Table S19 (Supplementary Material).

### 3.4 Summary

Contrary to Malt's results, we did not find an association between typicality ratings and H_2_O estimates provided by laypeople. We did instead replicate the result on the relation between typicality and centrality, suggesting laypeople's conceptualization of what counts as “water” is rooted in the importance of liquids in human life rather than in the liquid's chemical properties. In addition, and differently from Malt's findings, we found frequency ratings improved the predictive power on typicality ratings combined with centrality—hence further supporting the idea that experiences with the liquids are essential in the conceptualization of “water” (see S5 and S6 Supplementary Materials). These results are interesting because the mismatch between our data and data from Malt's study on the relation between H_2_O estimates and typicality further questions essentialist accounts of conceptual representation. At the very least, they suggest there might be variation in essentialist patterns for natural kinds, possibly caused by cross‐cultural and time‐related differences.

The pattern of results of chemists overall aligned with nonchemists’ ratings, with no relation between typicality and judged percentages of H_2_O, and a positive relation between typicality and centrality ratings. However, differently from nonchemists, we found that H_2_O estimates partially explain, together with centrality and frequency, which examples chemists think are typical for the category of “water.” This may suggest that chemists might implicitly incorporate more hidden and internal features, such as estimates of H_2_O, in their conceptual representation of “water.”

## 4 Experiment 3: The dimensions of water (exploratory)

Experiment 2 showed that laypeople's intuitions about water rest upon the centrality of liquids for human life, combined with the frequency with which participants interact with the liquids (consistently with Malt, 1994 and Chomsky, 1995). Estimated H_2_O percentages seemed to play little, if any, role in determining good exemplars of water. By contrast, chemists seemed to partly integrate H_2_O estimates in their conceptual representations of water along with centrality and frequency, suggesting a role for essentialist features in chemists’ conceptualization. Experiment 3 explores whether folk's understanding of water organizes along the same conceptual components of chemists’ understanding of water with similarity judgments, or whether chemists integrate in their semantic representation of water different features that might be related to the chemical composition of liquids.

### 4.1 Participants

#### 4.1.1 Nonchemists

Twenty participants took part in the study (*M*_age_ = 39.4; *SD*_age_ = 17.27; Age range = 23–70).

#### 4.1.2 Chemists

Twenty participants took part in the study (*M*_age_ = 38.55; *SD*_age_ = 12.27; Age range = 24–63).

### 4.2 Procedure

The questionnaires were composed of three sections. In the first section, we asked participants to judge how similar (1 = low similarity; 9 = high similarity) two examples of water were to each other. The order of the pairings was randomized across participants. The remaining two sections were identical to those of the previous experiments.

### 4.3 Materials

From the original list of water examples (see Table S1, Supplementary Appendix, Supplementary Material), we selected 20 items, 16 of which were identical to Malt's study and four arbitrarily chosen from our water examples—in line with the original study. We then created all 190 possible pairs resulting from the combination of the entire set of items to be presented to participants. The complete list of items is given in Table S2, Supplementary Appendix (Supplementary Material). In the original version of the task with English‐speaking participants, water examples were embedded into sentences. However, since there is no information about sentence construction, we reasoned that differences in linguistic context may have biased the results, making some features more salient than others—although we are aware that decontextualizing words is not unproblematic, as it may conceal important interpretive aspects. So, differently from the preregistration plan, we decided to refrain from embedding water examples in sentences and simply present participants with pairs of water examples to be rated.

### 4.4 Results

**Fig. 2:**
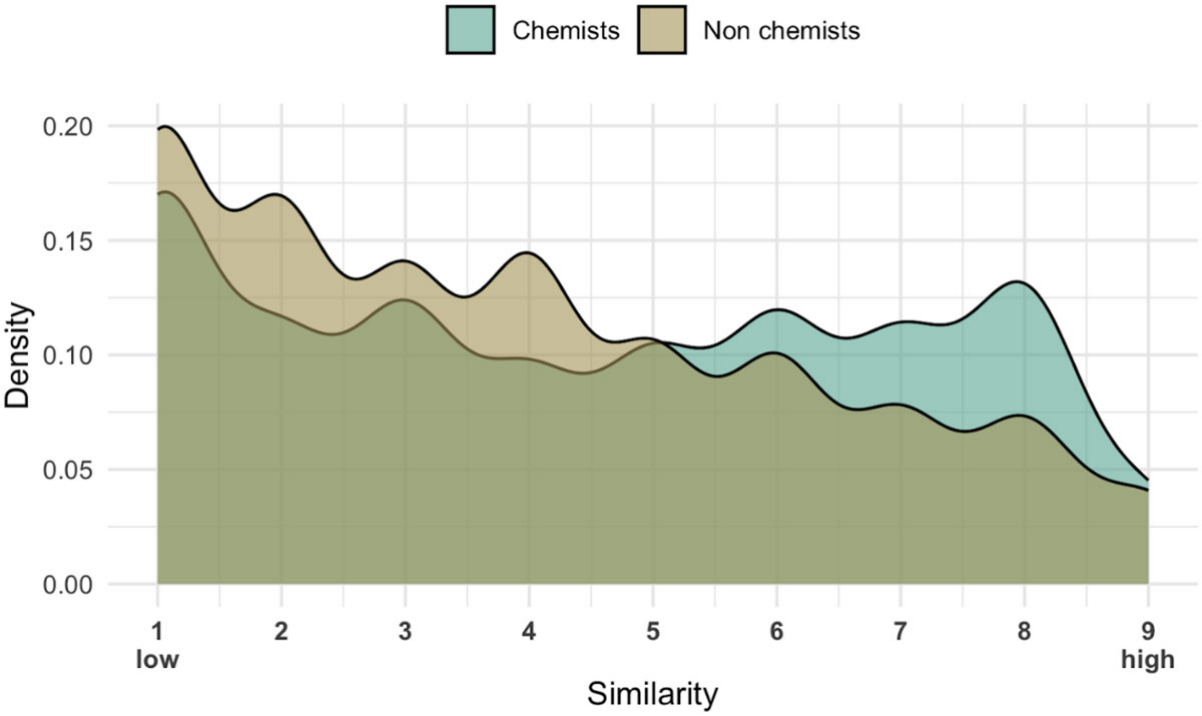
Distribution of similarity ratings for water examples provided by chemists and laypeople.

Both groups’ similarity ratings exhibited excellent internal consistency (*α* = .99). Chemists’ and laypeople's similarity ratings did not differ significantly, $\chi^{2}$ (1) = 1.201, *p* = .273, and were positively correlated, *t*(188) = 21.776, *p* < .001, *r* = .84, although chemists overall provided slightly higher similarity ratings than laypeople (*M* chemists = 4.51, *SD* = 2.56; *M* nonchemists = 3.94; *SD* = 2.40, see Fig. 2).

**Fig. 3:**
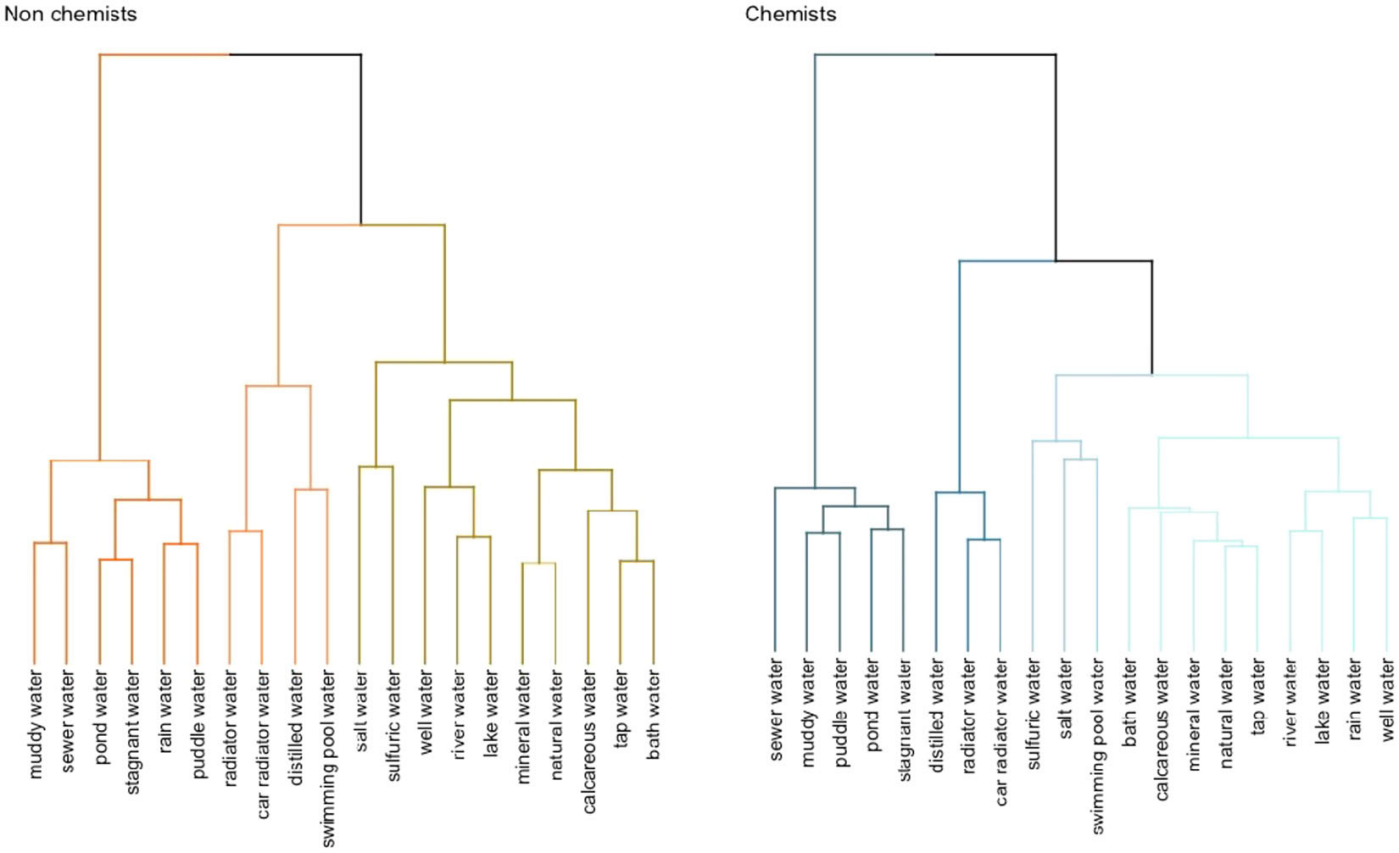
Dendrograms depicting (dis)similarity judgments of laypeople and chemists for (translated) water examples. In the dendrograms, more similar items are grouped together close to each other.

Both datasets showed good clusterability tendencies (nonchemists *H* = .63; chemists *H* = .66). Fig. 3 presents the dendrograms resulting from the dissimilarity matrices composed of laypeople and chemists’ similarity ratings.

The clusters showed both similarities and differences. In both groups, we found a cluster mostly encompassing “dirty” waters (e.g., *muddy water*, *pond water*, *puddle water*), with nonchemists including in this cluster also *rain water* (leftmost clusters). We also found a cluster encompassing types of water that might be relevant in everyday life for both groups (rightmost clusters), that shared the following exemplars: *bath water*, *tap water*, *calcareous water*, *natural water*, *mineral water*, *lake water*, *river water*, and *well water*. However, whereas nonchemists included *sulfuric* and *salt* water in this cluster, this was not the case for chemists, who instead included *rain water* in this cluster.

Notably, chemists grouped together in a separate cluster *swimming pool water*, *salt water*, and *sulfuric water*—possibly due to the presence of other well‐known chemical substances in these exemplars^3^ (i.e., Cl_2_ for swimming pool water, NaCl for salt water, S for sulfuric water). Indeed, among the entire list of exemplars, those are the only ones for which the solute of the solution is easily identifiable. The last nonchemists cluster contains types of water that might be interpreted as serving some sort of function (*swimming pool water*, *distilled water*, *car radiator water*, and *radiator water*), similarly to the remaining chemists’ cluster (*distilled water*, *car radiator water*, and *radiator water*).

Overall, these data partially align with those reported in Malt (1994). In fact, we also found an overarching distinction between “dirty” and more “domestic” waters holding across the two samples. Looking at smaller clusters, we can also notice the more fine‐grained distinction described by Malt between “outdoor” and “indoor” waters in the first clusters of both groups, with nonchemists grouping together *bath*, *tap*, and *calcareous* water; *natural* and *mineral* water; and *lake*, *river*, *and well* water. Likewise, chemists grouped together *tap*, *natural*, and *mineral* water, highlighting the “drinkable” character of this cluster; and *well*, *rain*, *lake*, and *river* water, representing the “outdoor” component.

### 4.5 Summary

Altogether, at least three prominent conceptual dimensions underlie laypeople's and chemists’ representations of water. These hinge on domestic, cleanliness, and functional aspects of water. So, although the data collection took place almost 30 years after the original study, and some of the linguistic examples were tailored to our Italian sample, our findings indicate that certain conceptual dimensions explain quite consistently the semantic organization of the water domain throughout time and across these two Western samples.

Nonetheless, our data also reveal fine‐drawn conceptual nuances that seem to be determined by expertise. Indeed, the most striking differentiation between the two dendrograms (i.e., the chemists cluster composed of *sulfuric*, *salt*, and *swimming pool water*) suggests information concerning the internal structure of specific kinds of water is more salient for chemists compared to laypeople.

## 5 Experiment 4

So far, data from laypeople indicate that folk conceptualizations of water extend to liquids containing varying percentages of H_2_O and other elements. While overall this was also true for chemists, their response pattern provided some evidence in favor of a stricter conception of “water,” perhaps pointing to more essentialist views of the meaning of “water” emerging from different experiences with the liquids and their constituents. Taken together, findings from laypeople and chemists do not offer conclusive evidence on the possible coexistence of two senses of “water” (i.e., one more general, including liquids with varying compositions, and one more strict referring only to “pure” water). Furthermore, it might be speculated that if these two senses coexist, they would be differently embraced by participants depending on their chemical expertise—such that chemists should be stricter about the meaning of “water” compared to laypeople. Experiment 4 aims to address these questions using sentence acceptability judgments, which tested intuitions of laypeople and chemists about the composition of liquids.

In Experiment 4, Part 1, participants were asked to judge the acceptability of sentences of the kind “X is a type of water” and “X is only partly water,” featuring water and nonwater examples. We hypothesized that if expertise does not affect the conceptualization of “water,” both laypeople and chemists would judge water examples to be types of water, but not to be only partly water. In addition, both laypeople and chemists would judge nonwater examples to be only partly water, but not to be types of water (Malt, 1994). By contrast, if expertise affects the conceptualization of “water,” both laypeople and chemists would still judge nonwater examples to be only partly water, but not to be types of water. Importantly, though, laypeople would judge water examples to be types of water, but not to be only partly water, whereas chemists would also judge water examples not to be types of water, but to be only partly water.

In Experiment 4, Part 2, participants were asked to judge the acceptability of sentences of the kind “X is a type of water” and “X is mostly but not entirely water,” featuring water and nonwater examples. We hypothesized that if expertise does not affect the conceptualization of “water,” both laypeople and chemists would judge nonwater examples to be mostly, but not entirely, water (Malt, 1994). By contrast, we specified in the preregistration that if expertise affects the conceptualization of “water,” chemists would not judge nonwater examples to be mostly, but not entirely, water, but we believe it is clear that the correct prediction would have been—similarly to that of Experiment 4, Part 1—that chemists would judge both water and nonwater examples as being mostly but not entirely water.

### 5.1 Experiment 4, Part 1: “X is a type of water” and “X is only partly water”

#### 5.1.1 Participants

##### 5.1.1.1. Nonchemists

Forty‐eight participants (*M*_age_ = 32.41; *SD* = 12.49, Age range = 22–75) took part in the study.

##### 5.1.1.2. Chemists

Sixty‐two participants (*M*_age_ = 39.96; *SD*_age_ = 12.47, Age range = 24–68) took part in the study.

#### 5.1.2 Materials

We randomly selected 18 water examples with six examples taken from each third of the distribution of judged percentages of H_2_O (retrieved from laypeople's estimates of Experiment 1, Part 2; see Malt, 1994). We also selected 18 nonwater examples with H_2_O estimated percentages within the H_2_O range of water examples and taken from each third of the distribution. Three stimulus sets were created and distributed across three groups of participants per group (i.e., laypeople vs. chemists). On average, water examples had comparable estimated H_2_O percentages across the three lists (list 1 *M* = 72.63; *SD* = 9.91; list 2 *M* = 72.60; *SD* = 6.34; list 3 *M* = 72.11; *SD* = 5.84). Likewise, nonwater examples also had comparable estimated H_2_O percentages across the three lists (list 1 *M* = 61.64; *SD* = 11.52; list 2 *M* = 61.17; *SD* = 8.77; list 3 *M* = 60.87; *SD* = 9.04). Each stimulus set is composed of 6 water examples (*n* = 2 per each third of the estimated H_2_O distribution) presented twice (once for each sentence type), 6 nonwater examples (*n* = 2 per each third of the estimated H_2_O distribution) presented twice (once for each sentence type), and 48 filler sentences. We created 96 filler sentences overall (half for each type of sentence) containing other substances (e.g., “oat milk is a type of milk”; “a towel is only partly cloth”). Within these sentences and across types of sentences, half (*n* = 24) was designed to be answered “yes,” and the other half (*n* = 24) was designed to be answered “no.” Finally, within this distinction, half fillers (*n* = 12) contained the name of the substance in the sentence, while the other half did not contain the name of the substance. The three lists only differ in the water and nonwater examples they comprise. The complete list of stimuli is available at https://osf.io/ycqan.

#### 5.1.3 Procedure

The questionnaires were composed of three sections. In the first section, participants were separately presented with the two types of questions (“X is a type of water” and “X is only partly water”) in two randomly presented blocks, along with filler sentences, and asked to decide whether the sentence was acceptable (“acceptable”) or not (“not acceptable”). Sentences were randomized within blocks. The last two sections of the questionnaire were identical to those of the previous experiments.

#### 5.1.4 Results

**Fig. 4:**
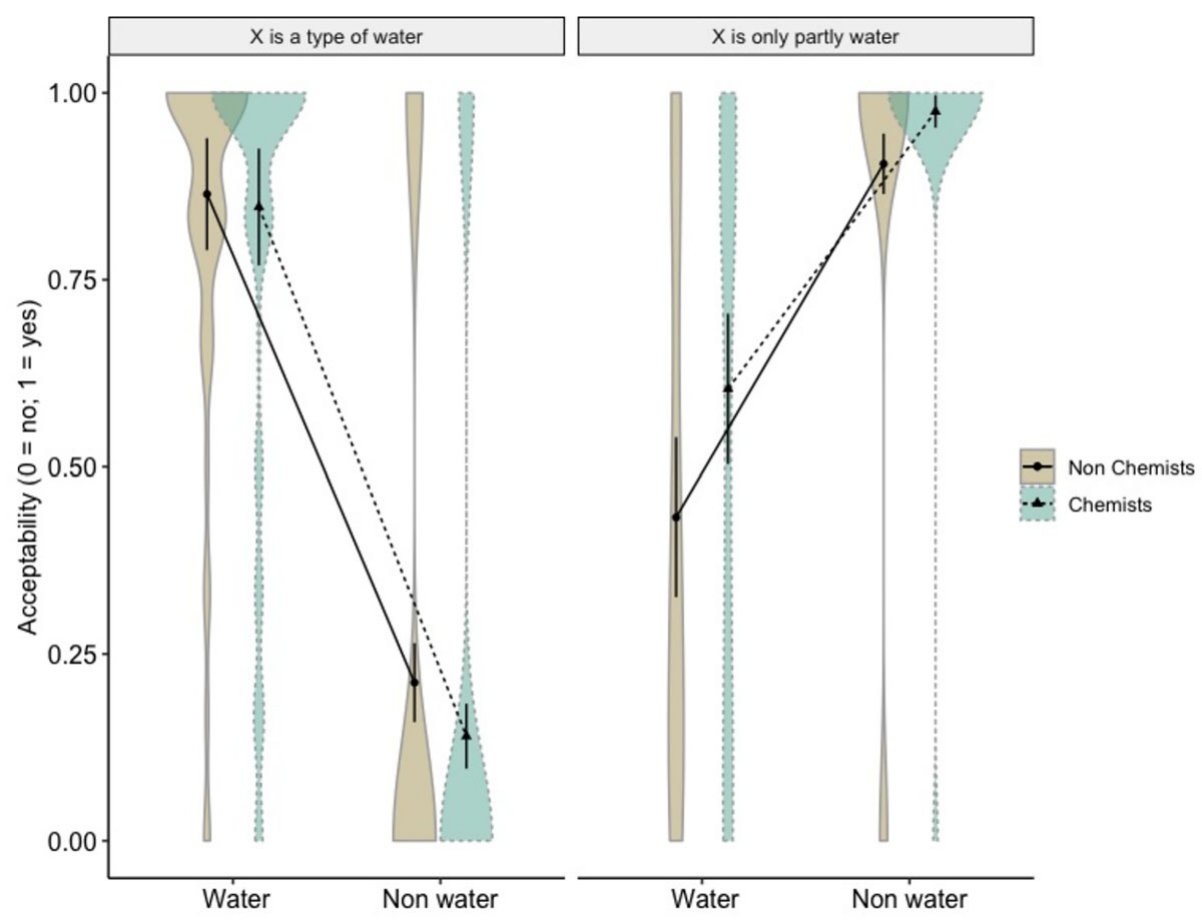
Acceptability responses distribution for water and nonwater examples to statements of the type “X is a type of water” and “X is only partly water” of nonchemists and chemists (Experiment 4, Part 1). Responses are given as predicted probabilities on the y‐axis.

We found a significant three‐way interaction between Group, Liquid Type, and Task (type vs. partly), $\chi^{2}$(1) = 4.861, *p* = .027, *b* = 1.08, 95% CI [0.12, 2.05]. We also found a significant two‐way interaction between Group and Task, $\chi^{2}$ (1) = 28.198, *p* < .0001, *b* = −1.92, 95% CI [−2.69, −1.14] and between Liquid Type and Task, $\chi^{2}$ (1) = 516.058, *p* < .0001, *b* = −6.78, 95% CI [−7.54, −6.01]. No other main effect reached significance, all *p*_s_ > . 14. Post‐hoc contrasts with Tukey's adjustment showed—contrary to our expectations—that laypeople and chemists were equally likely to think that water examples were “types of water,” *p* = .630, as they were for nonwater examples, *p* = .076. Instead, as predicted by the hypotheses, chemists were more likely than laypeople to think that both water, *z* = 2.701, SE = 0.257, *p* = .006, and nonwater examples, *z* = 3.742, SE = 0.378, *p* = .0002, were “only partly water” (see Fig. 4).

### 5.2 Experiment 4, Part 2: “X is mostly, but not entirely water”

#### 5.2.1 Participants

##### 5.2.1.1 Nonchemists

Forty‐six participants (*M*_age_ = 33.45; *SD*_age_ = 12.34, Age range = 23–64) took part in the study.

##### 5.2.1.2 Chemists

Forty‐nine participants (*M*_age_ = 37.97; *SD*_age_ = 12.62, Age range = 21–74) took part in the study.

#### 5.2.2 Procedure and design

The procedure and materials were the same as Experiment 4, Part 1, except for some filler sentences that were changed to comply with the experimental task. Differently from Experiment 4, Part 1, the second block of the first session required participants to judge the acceptability of sentences of the type “X is mostly, but not entirely water.” Data analysis was carried out as for Experiment 4, Part 1 (for details, please see Appendix, Supplementary Materials).

#### 5.2.3 Results

**Fig. 5:**
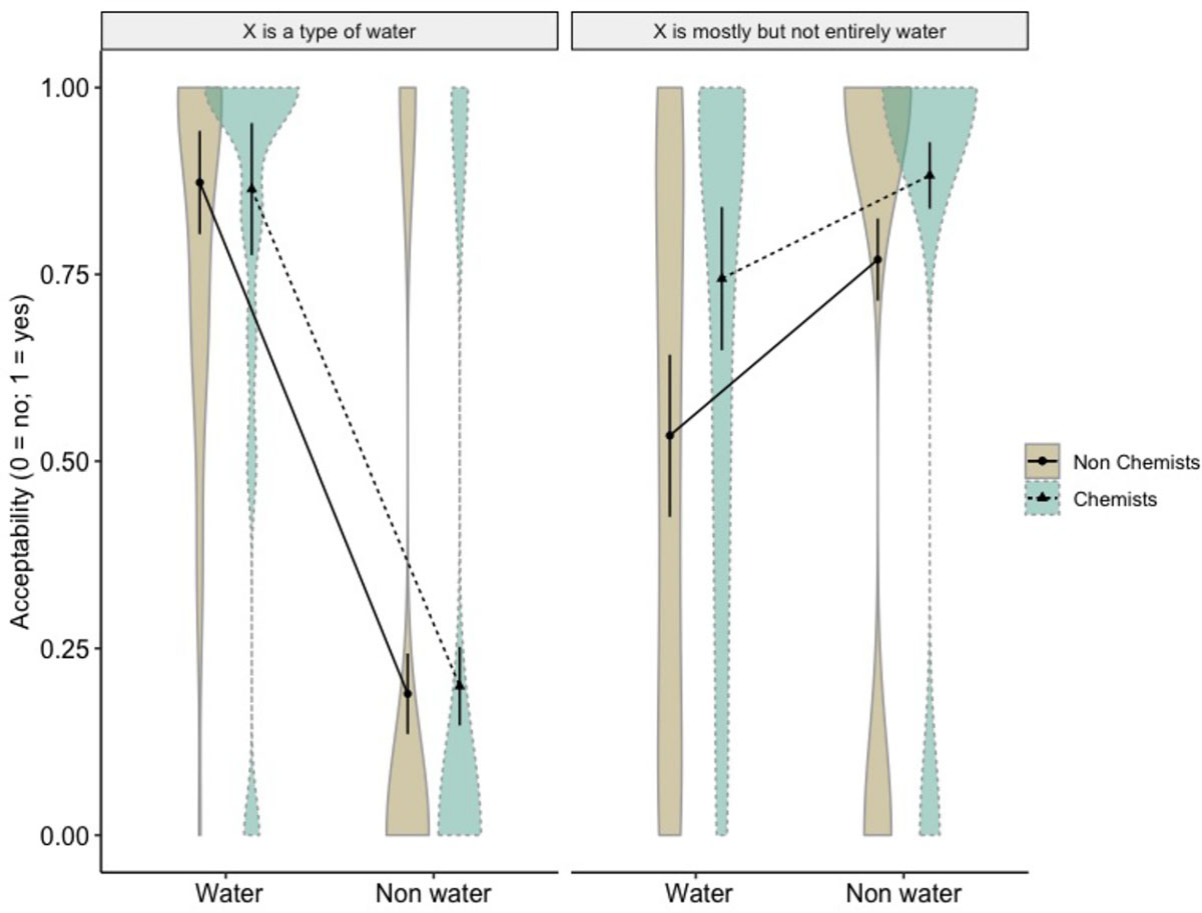
Acceptability responses distribution for water and nonwater examples to statements of the type “X is a type of water” and “X is mostly but not entirely water” of nonchemists and chemists (Experiment 4, Part 2). Responses are given as predicted probabilities on the y‐axis.

We found a significant main effect of Task, $\chi^{2}$ (1) = 22.36, *p* < .001, *b* = 3.41, 95% CI [2.91, 3.90]. We also found a significant interaction between Group and Task, $\chi^{2}$ (1) = 15.88, *p* < .001, *b* = −0.74, 95% CI [−1.39, −0.10], and between Liquid type and Task, $\chi^{2}$ (1) = 315.53, *p* < .001, *b* = −4.19, 95% CI [−4.84, −3.54]. No other effect or interaction reached significance, all *p*_s_ > .06. Post‐hoc contrasts showed that overall, chemists were more likely to think that both water and nonwater examples were “mostly, but not entirely water” compared to nonchemists, *z* = 3.149, SE = .276, *p* = .001. There was instead no difference in judgments of sentences of the sort “X is a type of water” across the two groups, *p* = .982. In addition, nonwater examples were overall less likely to be judged as “types of water” than water examples, *z* = −3.407, SE = 0.972, *p* = .0007, whereas there was no difference between the two types of liquids in sentences of the sort “X is mostly, but not entirely water,” *p* = .297 (see Fig. 5).

#### 5.2.4 Summary

All in all, these data provide support for the hypothesis that expertise affects the conceptualization of water. In fact, we found that chemists were more likely than laypeople to judge sentences of the sort “X is only partly water” and “X is mostly but not entirely water” acceptable both for nonwater examples and—more critically—for water examples. This suggests that chemists might have a stricter conceptualization of what counts as “water” than nonchemists, and this conceptualization might partially overlap with that of “H_2_O,” as even water examples were interpreted as being mixtures (i.e., only partly and mostly but not entirely) of water and other ingredients. These data further suggest that chemists exhibit an increased degree of category fuzziness (Lakoff, 1973) when evaluating water. They classify water examples as belonging to the category of water, yet simultaneously acknowledge that these examples are only partially or predominantly, but not entirely, water. This finding indicates that chemists’ semantic representation of water encompasses both a coarser idea of a liquid—such as the one exhibited by laypeople—and its chemical identity as H₂O, with these conceptualizations becoming salient when explicitly examined.

## 6 Experiment 5: Abstractness ∼ concreteness ratings for “water” and “H_2_O”

There is now evidence that categories might be conceptualized as more abstract or more concrete across different sociocultural groups, depending on life experiences and cultural parameters (Majid et al., 2018; Mazzuca et al., 2020; Mazzuca et al., 2024; Villani et al., 2022). Experiment 5 explores whether there are differences in ratings of abstractness ∼ concreteness for “H_2_O” and “water” across laypeople and chemists. This is interesting because, if essentialist views are correct, then there should be no difference in ratings of abstractness ∼ concreteness for the two concepts across the board—as the two labels would ideally point to the same referent. Instead, if variation exists, we should find differences across the two groups. Specifically, chemists might think of H_2_O in more concrete terms compared to laypeople, given their extensive experience with the compound. Laypeople, on the other hand, who may perceive H_2_O as less salient and more psychologically distant (Mazzuca, Falcinelli, Michalland, Tummolini, & Borghi, 2022) compared to “water” might perceive “H_2_O” as more abstract than chemists.

### 6.1 Participants

#### 6.1.1 Nonchemists

Ratings were provided by a total of 276 participants (*M*_age_ = 33.05; *SD* = 13.13, Age range = 18–75).

#### 6.1.2 Chemists

Ratings were provided by a total of 277 participants (*M*_age_ = 39.77; *SD* = 13.04, Age range = 21–81). It has to be borne in mind that—as previously noted throughout the text—these data were collected after each main task of all the experiments composing this study, and so the sample of participants described here corresponds to the entire sample enrolled in the study.^4^

### 6.2 Procedure and materials

Abstractness ∼ concreteness ratings were collected after each main task described in the previous sections. At the end of each experimental task, participants were asked to rate on a 7‐point Likert scale whether “H_2_O” and “water” are concrete or abstract (1 = completely concrete; 7 = completely abstract). The order of the two questions was randomized across participants within each study.

### 6.3 Results

We found a main effect of Group, $\chi^{2}$ (1) = 63.624, *p* = .004, a main effect of Concept, $\chi^{2}$ (1) = 19.776, *p* = .021, and a significant interaction between Group and Concept, $\chi^{2}$ (1) = 22.954, *p* = .004. Post‐hoc contrasts showed that laypeople rated both “water” *z* = 2.516, *p* = .011, and “H_2_O,” *z* = 8.401, *p* < .0001, as more abstract compared to chemists. In addition, laypeople rated “H_2_O” as significantly more abstract than “water,” *z* = 6.263, *p* < .0001, whereas there was no difference between the two concepts in chemists’ ratings, *p* = .351 (see Fig. 6).

**Fig. 6:**
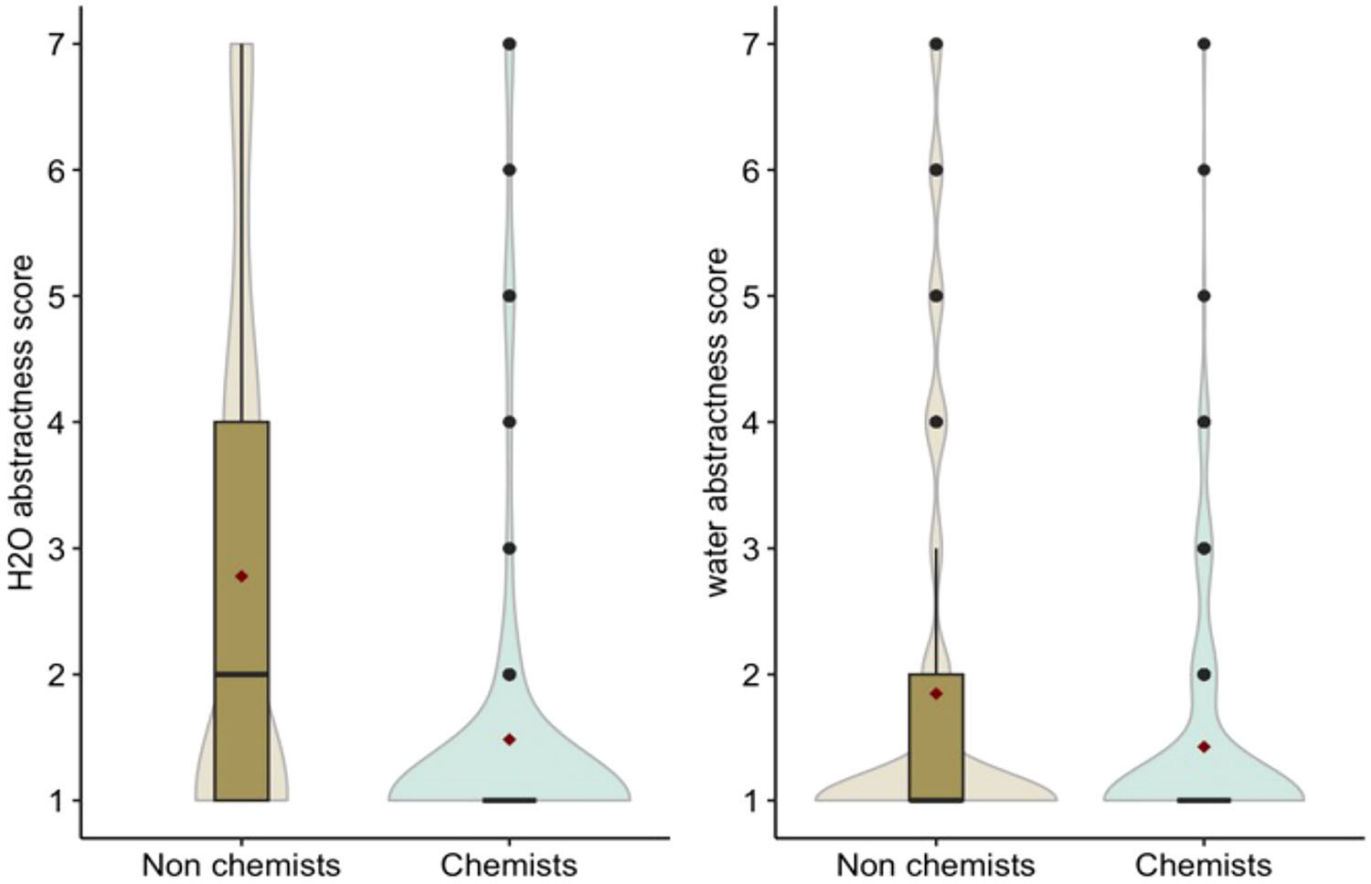
Abstractness∼concreteness ratings for “H_2_O” (leftmost panel) and “water” (rightmost panel) provided by laypeople and chemists. In the boxplots, red dots indicate mean values, black bold horizontal lines the median, and vertical extremes of the boxplots represent minimum and maximum values in the data. The boxes’ length shows the interquartile range, with the upper side representing the 75th percentile and the bottom side the 25th percentile. Violins represent the distribution of data, and black dots represent outliers.

Overall, then, we found that although according to essentialist perspectives “H_2_O” and “water” should point to the same meaning, the extent to which these concepts are considered abstract varies as a function of participants’ background. Specifically, laypeople thought “H_2_O” was consistently more abstract than “water,” whereas for chemists, the two labels were equally concrete—perhaps suggesting two overlapping conceptual representations. Previous research shows that expertise can interact with abstraction, affecting the hierarchical structuring of categories across experts and novices (Tanaka & Taylor, 1991). Our findings cast new light on the relation between expertise and categorization, suggesting that not only concepts abstraction, but also abstractness (for a discussion on the difference between abstraction and abstractness, see Borghi & Binkofski, 2014, Reilly et al., 2024) can be influenced by differing levels of expertise—hence, different experiences—with specific entities.

## 7 General discussion

Across five studies, we found that conceptualizations of “water” can be subtly shaped by expertise. More specifically, our data provide evidence favoring a nuanced account of psychological essentialism, showing that essentialist patterns of conceptualization may be flexibly modulated by different experiences, thereby reconciling psychological essentialism and conceptual flexibility. When asked to estimate H_2_O percentages of liquids like tea or juices (Experiment 1, Part 2), laypeople and chemists provide comparable and rather accurate estimates. Perhaps unsurprisingly, chemists’ estimates are slightly more conforming to the “true” H_2_O percentages of liquids than those provided by laypeople. Experiment 2 showed that what essentialist views would propose as the true essence of water—that is, H_2_O amount—did not account in and of itself for which liquids are considered typical examples of water across the two groups. Instead, we found that, overall, liquids that are central to human experiences, as well as liquids that are more frequently encountered, are also thought of as more typical examples of water. Interestingly, though, estimated percentages of H_2_O had a marginal effect on chemists’ typicality judgments, together with centrality and frequency, while this was not the case for laypeople. Experiment 3 also confirmed the substantial role of human experiences in the representation of the semantic domain of “water.” In fact, both laypeople's and chemists’ similarity judgments converged on a configuration evidencing three main underlying semantic components, broadly related to everyday interactions with the liquids. Remarkably, chemists’ dendrogram comprised one additional cluster including three examples whose chemical composition includes salient well‐known elements (Cl_2_ for swimming pool water, NaCl for salt water, S for sulfuric water) besides H_2_O. It might be speculated that in these cases, “impurities” like these further elements are indeed essential components determining the specific ontological status of these compounds (in line with Abbott, 1999), so that, at least implicitly, chemists tend to distinguish them from other water examples. Findings from Experiment 4 exhibited a composite pattern—suggesting caution in adopting all‐or‐nothing explanations. Indeed, a strong essentialist perspective would have predicted that chemists should not find acceptable sentences in which examples are said to be “types of water,” as the linguistic label “water” should only apply to pure H_2_O—and, therefore, talking about types of H_2_O should be considered implausible. Our data speak to a more nuanced and multifaceted perspective. In both versions of Experiment 4 (Parts 1 and 2), chemists and laypeople were equally likely to find acceptable “type of water” sentences featuring water examples. However, when asked to reason in compositional terms about the liquids, the two groups showed intriguing differences, with chemists exhibiting a narrower conception of “water.” Not only were chemists more likely than laypeople to think that nonwater examples are “only partly” water, but this was also true for their judgments on water examples. In addition, chemists were overall more likely than laypeople to think that all the liquids (water and nonwater examples) were “mostly but not entirely water.” In some sense, then, chemists’ H_2_O thresholds for the inclusion of liquids in the category of “water” seem to be so high when targeted explicitly in compositional terms that might also prevent water examples from being included in the category (see LaPorte, 1998).

Taken together, our findings align well with research on the relation between expertise and conceptual representation, adding further insights. Overall, we found that chemists’ conceptualization of “water” partly relies on internal, chemical properties of liquids. This is consistent with studies on other domains showing that experts’ categorizations exploit different knowledge structures (Tanaka & Taylor, 1991). Compared to laypeople or novices who mainly base their judgments on surface‐level information, experts might ground their judgments on functional features that are less perceptually obvious—and that contribute to a finer parsing of subcategory distinctions (Wing et al., 2022). For instance, wine and coffee experts tend to use more source‐based terms (e.g., *vanilla*) for describing wines and coffees compared to novices, who instead use more evaluative terms (e.g., *nice*, Croijmans & Majid, 2016). It is worth noting that, while previous studies in this area indicate that different levels of expertise impact both taxonomic structures (i.e., abstraction) and the relevance of specific hidden dimensions in conceptualization, here we found that abstractness, too, can be influenced by expertise. Interestingly, our results depart from those reported in a recent cross‐linguistic study targeting the conceptual representation of the waterbodies domain across laypeople and experts (Purves, Striedl, Kong, & Majid, 2023). When asked to rate waterbodies in terms of sensory, motor, and emotional components, experts and laypeople of both linguistic groups (i.e., English and German) did not differ, with ratings substantially aligned. This underscores that the impact of expertise on conceptualizations might vary as a function of the specific semantic dimension targeted, as well as on the task being performed.

Our study also has clear implications for the literature on psychological essentialism. Traditional accounts of essentialism propose that the essence shared by category members is represented by a “placeholder” (Gelman, 2003; Medin & Ortony, 1989)—that is, some causal essence that stitches category members together but can be unknown to people. In keeping with Putnam (1975) and Kripke (1980), Gelman (2003) suggests that this placeholder can be filled in and refined with the acquisition of scientific knowledge. Along these lines, once discovered that water is H_2_O, people would hold that H_2_O is the true essence of water, a perspective also known as “scientific essentialism” (Rose & Nichols, 2019). While Malt's (1994) results with laypeople contradict this approach, our findings with chemists provide partial support to this idea, when interpreted in light of the evidence about psychological essentialism variability. Indeed, our results showed that not only categories might be more or less essentialized as a function of linguistic and cultural context (Rhodes & Gelman, 2009; Diesendruck et al., 2013; Smyth et al., 2017; Machery et al., 2023), but also as a function of differing levels of expertise—further stressing the idea that the saliency of a category in a given context can partially explain essentialist thinking (Pauker, Tai, & Ansari, 2020). In fact, there is no doubt that laypeople are also aware that water is H_2_O, but this information is likely less relevant in their everyday life compared to chemists. This is also consistent with recent accounts, suggesting there is no uniform answer to the question of whether reference for natural kinds is solely based on functions or on internal properties (or on descriptive vs. causal‐historical features, Devitt & Porter, 2021). For instance, Tobia, Newman, and Knobe (2020) found that laypeople were more likely to rely on superficial properties for judgments about category membership of natural kinds when presented with a legal context, whereas they were more likely to rely on deep structural properties when presented with a scientific context. So, these concepts might have two distinct “senses,” that is, a descriptive and a causal‐historical one, and these distinct “senses” are differently retrieved depending on the situation (see also Genone & Lombrozo, 2012; Haukioja, Nyquist, & Jylkkä, 2021). More generally, it may be speculated that the lay term “water” is more ambiguous for chemists than for laypeople (Abbott, 1997). As Devitt and Porter (2021) put it, “When doing science, they [chemists] regularly use ‘water’ to refer to ‘deep‐causal‐water’, but when they leave the laboratory and talk to the folk, they regularly use it to refer to ‘superficial‐water’. […] The folk may differ from the chemists in not having these two regular uses, such that ‘water’ is ambiguous in the chemists’ idiolect but unambiguous in the folks’.” (Devitt & Porter, 2021, p. 20). Overall, then, our work shows that conceptual representations vary depending on expertise, and so does psychological essentialism—even within the same sociocultural context.

Finally, our data also add layers to the literature on concepts’ abstractness ∼ concreteness. Research in this area traditionally focused on the distinction between abstract and concrete concepts to account for differential patterns of representation and processing (Paivio, 1990), showing that more abstract concepts are more difficult to elaborate (Schwanenflugel, 1991), are acquired later (Gilhooly & Watson, 1981), are perceived as more difficult and more psychologically distant (Mazzuca et al., 2022), and are thought to be more variable across contexts and cultures (Gentner & Asmuth, 2019; Malt & Wolff, 2010; Thompson, Roberts, & Lupyan, 2020; Borghi & Mazzuca, 2023). Recent findings suggest that the extent to which a category is perceived as abstract or concrete can vary depending on cultural, linguistic, and sociodemographic components (Majid et al., 2018; Mazzuca et al., 2020; Banks et al., 2023). To illustrate, Mazzuca et al. (2024) found that the concept of “gender” is represented in more abstract terms by Italian speakers, whereas Dutch speakers focus on more concrete, biological terms. On top of that, results from an explicit task tackling essentialist and constructivist beliefs about gender across the two groups showed that Dutch participants endorsed more essentialist beliefs about gender than Italian participants. Along similar lines, here, we found that while laypeople rated “H_2_O” as being consistently more abstract than “water,” chemists judged the two concepts as being equally concrete—and significantly more concrete compared to laypeople. This is interesting because universalist perspectives on meaning would have predicted less variation in the representation of concrete, natural concepts like “water” (Thompson et al., 2020). Against this backdrop, natural kind concepts should exhibit less conceptual variation than more abstract concepts, as they would reflect stable and defined perceptual properties of the environment. Our study showed that even an element that is so essential to human life such as water varies in conceptual abstractness across people with different expertise—further suggesting that abstract or concrete components of a concept might be more salient depending on sociocultural context and long‐term experiences (Barsalou, Dutriaux, & Scheepers, 2018; Borghi et al., 2019; Majid et al., 2018; Yee & Thompson‐Schill, 2016; Mazzuca et al., 2024).

## 8 Limitations

The present study confines itself to Italian speakers sampled through an online platform, therefore, constraining its generalizability to a Western, industrialized, highly educated, and digitally connected cultural milieu (Ghai et al., 2025). However, differently from the original study, we aimed at diversifying our sample of lay participants from psychology undergraduates. This being said, we need to highlight that our sample of laypeople does not constitute a single homogeneous group. There might be individual differences related to educational background or other factors that we did not take into account in this work. For instance, recent research shows that education level might impact conceptual representations (Verheyen, Droeshout, & Storms, 2019). Our sample of chemists was, on average, more educated than our sample of laypeople (see S1.1, Supplementary Material). More specifically, across the experiments, the two samples differed on the highest and lowest extremes of education levels (e.g., high school and PhD). However, there was no difference between the two groups with respect to medium‐higher education levels (i.e., Bachelor's and Master's Degree). Consequently, a more rigorous test may be required to specifically target nonchemists with higher education in other disciplines, thereby ensuring more comparable results. Additionally, our samples were not always balanced in terms of gender, an aspect that has been shown to affect categorization patterns (Stukken, Verheyen, & Storms, 2013).

To assess the impact of expertise on the conceptualization of “water,” we specifically targeted experienced chemists. This was mainly motivated by the theoretical account we sought to test, that is, psychological essentialism, which stresses the importance of the underlying chemical structure of natural kinds. Yet, there may be other scientific fields (e.g., hydrology, biology) or relevant expertise (e.g., water sommelier) that can equally affect the conceptual representation of the domain in unexpected ways. Finally, while we attempted to replicate Malt's (1994) procedure and materials as closely as possible, our study presents some differences that are noteworthy for comparisons with the original study and for future replications. First, since stimuli are generated from a free‐listing task by participants (Experiment 1, Part 1), these are necessarily historically and culturally bounded. Second, given that sentences for Experiments 3 and 4 were not available in the original study, we only used decontextualized liquids in Experiment 3 and crafted a new set of Italian sentences following the indication provided in Malt (1994) for Experiment 4 (see OSF repository).

## 9 Conclusions

To conclude, our work shows that the representation of water cannot be narrowed down to its chemical composition, but it can vary as a function of expertise. These findings contribute to a wider understanding of conceptual representations, showing that these can be contextually shaped by multiple components (Yee & Thompson‐Schill, 2016), including expertise (Wing et al., 2022; Tanaka & Taylor, 1991; Croijmans & Majid, 2016; Croijmans, Speed, Arshamian, & Majid, 2020; Villani et al., 2022). Furthermore, our results also suggest concepts representation cannot be understood as either essentialized or culturally constructed (see also Tobia et al., 2020; Devitt & Porter, 2021), nor as uniquely concrete or abstract (Mazzuca et al., 2024; Banks et al., 2023). This points to the importance of considering variability along multiple dimensions—including educational background and expertise—as a constituent element of conceptual representations, therefore, questioning universalist explanations of psychological phenomena (Majid, 2023). Finally, our research has implications for cognitive science overall. We provide new experimental support to Malt's (1994) findings with laypeople with a replication tailored to the linguistic specificity of the Italian context, showing that previous results obtained from a sample of convenience of US students replicate across another Western culture and over time—thereby speaking to replicability and generalizability issues that have been raised in psychology over the last decade (Open Science Collaboration, 2015; Munafò et al., 2017; Youyou, Yang, & Uzzi, 2023; Blasi, Henrich, Adamou, Kemmerer, & Majid, 2022).

## Author contributions

**CM**: Conceptualization; data curation; methodology; investigation; formal analysis; visualization; funding acquisition; writing—original draft. **MA**: Data curation; project administration; writing—review and editing. **IF**: Writing—review and editing. **CF**: Writing—review and editing. **AMB**: Conceptualization; resources; supervision; funding acquisition; writing—review and editing.

## Funding information

This work was supported by Sapienza's Starting Research Grant (“Progetti Avvio alla Ricerca”) n. AR2231888CC1C73D led by CM and by PNRR funds, PE8 ‐ AGE‐IT ‐ Spoke 4: Healthy aging. Project line led by AMB: “Older adults’ relationship with the natural environment to enhance their cognitive performances and promote mental health.”

## Conflict of interest statement

The authors declare no conflict of interest.

## Ethical approval statement

Ethical approval for all the experiments reported in the paper was provided by the Ethics Committee of the Department of Dynamic, Clinical Psychology and Health (Prot. n. 0000754) of Sapienza University of Rome.

## Supporting information



Supplementary Materials

## Data Availability

This study was preregistered at https://osf.io/hrvjw/?view_only=f9532b6932a14b03a8b609a33a0741a2. Data, analyses scripts, and materials are publicly available at https://osf.io/ycqan/?view_only=e3edaf460d0f46d7a808f6f24dd0c463.

## Notes

1 Psychological essentialism is the view that essences are a heuristic or mental shortcut and differs from metaphysical essentialism according to which objects have essences, or more precisely, (at least some) objects have (at least some) essential properties (Robertson, 2008). Metaphysical essentialism is a doctrine about objects in the world and their properties. At the core of the doctrine is the idea that an object O has a property P *essentially* when O must have P to be the object that it is. So, the features of an object are not merely the product of how we think or talk about the object but are features of the way the objects are in themselves. That is, in any possible world, for an object to be O, it has to possess P. For instance, for something to be water, it has to possess two hydrogen atoms and one oxygen atom. “Essentialims is […] a theory of the objective, context independent *de re* natures of objects.” (Paul, 2004, p. 170). Cognitive scientists have been mainly interested in psychological essentialism, as it taps into people's intuitions of what things in the world are rather than objective realities. In keeping with that, for the remainder of this article, we will be concerned with psychological essentialism—unless otherwise specified.

2 Malt's (1994) work specifically addressed Putnam's (1975) theory of reference, that is, “semantic externalism,” which is well‐exemplified by his Twin Earth thought experiment. In this hypothetical scenario, a liquid on another planet, identical to planet Earth, is said to have all the descriptive properties of water (e.g., tasteless, transparent), but is not made of H_2_O—its chemical composition is instead something very complex that is approximated as XYZ. This liquid is nonetheless called “water” by Twin Earth inhabitants. So, while Earthlings and their twins on Twin Earth both refer to the liquid as “water,” they are actually referring to two different liquids. This was meant to illustrate that descriptive parameters speakers associate with the referent are not enough for a proper determination of the referent. Rather, internal and essential features of the referents need to be taken into account to correctly grasp the determination of meaning, an account often also referred to as *causal‐historical*. This view is often contrasted to *descriptivist* accounts of meaning (e.g., Frege, 1892), which hold that the reference of a name is determined by the set of descriptions a speaker associates with it, whose content uniquely determines the name's referent. A similar debate pertains linguistics, where some have proposed that meanings can be accurately defined only when we possess some kind of scientific knowledge about the matter. So, for instance, Bloomfield (1933) argues that the ordinary meaning of the English word “salt” is “NaCl.” By contrast, Palmer (1976) maintains that there is no reason to believe that scientific definitions are linguistically more accurate than nonscientific ones and that “the precision of scientific definition serves the scientist's purpose, but is in no way related to human language.” (p. 54).

3 Pointed to us by one of the two independent chemists. One might think that this would also be true for “calcareous water,” but the general extension of the label can also be applied to a solution containing more than one solute dissolved in water, for example, potassium, sodium, and calcium carbonate.

4 Please also note that due to a technical problem, Experiment 4, Part 2, has been conducted twice as the sentence acceptability data gathered in the first data collection could not be used. Instead, we kept abstractness ∼ concreteness ratings—thus, motivating the discrepancy between the total number of participants mentioned in this section and that of the previous studies.
