# Supplementary material for: Water Is and Is Not H2O, Depending on Who You Ask: Conceptualizations of Water Vary Across Chemists and Laypeople
Source: Cogn Sci. 2025 Aug 5;49(8):e70094. doi: 10.1111/cogs.70094 (PMC12323298; doi:10.1111/cogs.70094)
Supplement: Supplementary file 1 — Supplementary Materials [file COGS-49-e70094-s001.docx]

**Water is and is not H_2_O, depending on who you ask.**

**Conceptualisations of water vary across chemists and laypeople**

**Supplementary Materials**

**S1. Demographic characteristics of participants across the studies**

*Note.* Tables 1 – 8 report the percentage of participants for each level of birth sex, gender, and scientific area (in columns), along with the total number of participants for each group (laypeople and chemists) for each study and—where relevant—each specific task.

**Table 1.** *Experiment 1 Part 1*

|  | **Water** | **Non-Water** |
| --- | --- | --- |
| Birth Sex |  |  |
| Male | 41.2 | 40.0 |
| Female | 52.9 | 60.0 |
| Intersex | 5.9 | // |
| Gender |  |  |
| Man | 35.3 | 33.3 |
| Woman | 52.9 | 46.7 |
| Queer | 11.8 | 13.3 |
| Trans | // | // |
| Other | // | 6.7 |
| Total *(N*) | 17 | 15 |

**Table 2.** *Experiment 1.1A*

|  | **Water** | **Non-Water** |
| --- | --- | --- |
| Birth Sex |  |  |
| Male | 53.3 | 6.7 |
| Female | 46.7 | 93.3 |
| Intersex | // | // |
| Gender |  |  |
| Man | 53.3 | 6.7 |
| Woman | 46.7 | 93.3 |
| Queer | // | // |
| Trans | // | // |
| Other | // | // |
| Total *(N*) | 15 | 15 |

**Table 3.** *Study 1 Part 2*

|  | **Water** | | **Non-water** | |
| --- | --- | --- | --- | --- |
|  | **Laypeople** | **Chemists** | **Laypeople** | **Chemists** |
| Birth Sex |  |  |  |  |
| Male | 17.4 | 47.8 | 45.5 | 34.8 |
| Female | 82.6 | 52.2 | 54.5 | 65.2 |
| Intersex | // | // | // | // |
| Gender |  |  |  |  |
| Man | 17.4 | 43.5 | 40.9 | 34.8 |
| Woman | 82.6 | 52.2 | 54.5 | 65.2 |
| Queer | // | 4.3 | // | // |
| Trans | // | // | 4.5 | // |
| Other | // | // | // | // |
| Scientific Area |  |  |  |  |
| Mathematical and Computer Sciences | 4.3 | // | 9.1 | // |
| Physical Sciences | // | // | 4.5 | // |
| Chemical Sciences | // | 100 | 4.3 | 100 |
| Earth Sciences | // | // | // | // |
| Biological Sciences | // | // | // | // |
| Medical Sciences | 21.7 | // | 22.7 | // |
| Agricultural and VeterinarySciences | // | // | 4.5 | // |
| Civil Engineering and Architecture | 4.3 | // | // | // |
| Industrial and Information Engineering | // | // | // | // |
| Antiquity, Philological-Literary, and      Historical-Artistic Sciences | // | // | // | // |
| Historical, Philosophical, Pedagogical,      and Psychological Sciences | 8.7 | // | 4.5 | // |
| Legal Sciences | // | // | 4.5 | // |
| Economic and Statistical Sciences | 4.3 | // | // | // |
| Political and Social Sciences | // | // | 4.5 | // |
| Not Applicable | 56.5 | // | 45.5 | // |
| Total (*N*) | 23 | 23 | 22 | 23 |

**Table 4.** *Study 1 Part 1A*

|  | **Water** | **Non-Water** |
| --- | --- | --- |
| Birth Sex |  |  |
| Male | 53.3 | 6.7 |
| Female | 47.7 | 93.3 |
| Intersex | // | // |
| Gender |  |  |
| Man | 53.3 | 6.7 |
| Woman | 47.7 | 93.3 |
| Queer | // | // |
| Trans | // | // |
| Other | // | // |
| Total *(N*) | 15 | 15 |

**Table 5.** *Study 2*

|  | **Typicality** | | **Centrality** | | **Frequency** | |
| --- | --- | --- | --- | --- | --- | --- |
|  | **Laypeople** | **Chemists** | **Laypeople** | **Chemists** | **Laypeople** | **Chemists** |
| Birth Sex |  |  |  |  |  |  |
| Male | 30.8 | 42.3 | 40.0 | 35.0 | 45.0 | 50.0 |
| Female | 65.4 | 57.7 | 55.0 | 65.0 | 55.0 | 50.0 |
| Intersex | 3.8 | // | 5.0 | // | // | // |
| Gender |  |  |  |  |  |  |
| Man | 30.8 | 42.3 | 40.0 | 30.0 | 45.0 | 50.0 |
| Woman | 69.2 | 53.8 | 55.0 | 70.0 | 55.0 | 50.0 |
| Queer | // | 3.8 | 5.0 | // | // | // |
| Trans | // | // | // | // | // | // |
| Other | // | // | // | // | // | // |
| Scientific Area |  |  |  |  |  |  |
| Mathematical and    Computer Sciences | // | // | 5 | // | // | // |
| Physical Sciences | // | // | // | // | 5 | // |
| Chemical Sciences | // | 100 | // | 100 | // | 100 |
| Earth Sciences | // | // | // | // | // | // |
| Biological Sciences | // | // | 5 | // | // | // |
| Medical Sciences | 11.5 | // | 5 | // | 5 | // |
| Agricultural and       VeterinarySciences | // | // | // | // | // | // |
| Civil Engineering        and Architecture | // | // | 5 | // | 5 | // |
| Industrial and       Information       Engineering | 3.8 | // | // | // | // | // |
| Antiquity,      Philological-      Literary, and      Historical-Artistic      Sciences | 7.7 | // | 10 | // | // | // |
| Historical,      Philosophical,      Pedagogical,      and Psychological      Sciences | 30.8 | // | 25 | // | 10 | // |
| Legal Sciences | // | // | // | // | 5 | // |
| Economic and      Statistical Sciences | // | // | 5 | // | // | // |
| Political and Social      Sciences | 3.8 | // | 5 | // | 10 | // |
| Not Applicable | 42.3 | // | 35 | // | 60 | // |
| Total (*N*) | 26 | 26 | 20 | 20 | 20 | 20 |

**Table 6.** *Study 3*

|  | **Laypeople** | **Chemists** |
| --- | --- | --- |
| Birth Sex |  |  |
| Male | 50.0 | 30.0 |
| Female | 50.0 | 70.0 |
| Intersex | // | // |
| Gender |  |  |
| Man | 50.0 | 30.0 |
| Woman | 50.0 | 70.0 |
| Queer | // | // |
| Trans | // | // |
| Other | // | // |
| Scientific Area |  |  |
| Mathematical and Computer Sciences | // | // |
| Physical Sciences | // | // |
| Chemical Sciences | // | 100 |
| Earth Sciences | // | // |
| Biological Sciences | // | // |
| Medical Sciences | // | // |
| Agricultural and VeterinarySciences | // | // |
| Civil Engineering and Architecture | // | // |
| Industrial and Information Engineering | 5 | // |
| Antiquity, Philological-Literary, and      Historical-Artistic Sciences | // | // |
| Historical, Philosophical, Pedagogical,      and Psychological Sciences | 50.0 | // |
| Legal Sciences | // | // |
| Economic and Statistical Sciences | 5.0 | // |
| Political and Social Sciences | // | // |
| Not Applicable | 40.0 | // |
| Total (*N*) | 20 | 20 |

**Table 7.** *Study 4*

|  | **Only partly** | | **Mostly but not entirely** | |
| --- | --- | --- | --- | --- |
|  | **Laypeople** | **Chemists** | **Laypeople** | **Chemists** |
| Birth Sex |  |  |  |  |
| Male | 35.4 | 38.7 | 37.0 | 42.9 |
| Female | 64.6 | 61.3 | 60.9 | 57.1 |
| Intersex | // | // | 2.2 | // |
| Gender |  |  |  |  |
| Man | 35.4 | 38.7 | 30.4 | 42.9 |
| Woman | 64.6 | 61.3 | 58.7 | 57.1 |
| Queer | // | // | 8.7 | // |
| Trans | // | // | 2.2 | // |
| Other | // | // | // | // |
| Scientific Area |  |  |  |  |
| Mathematical and Computer Sciences | 2.1 | // | 2.2 | // |
| Physical Sciences | // | // | 4.3 | // |
| Chemical Sciences | // | 100 | // | 100 |
| Earth Sciences | // | // | // | // |
| Biological Sciences | 2.1 | // | // | // |
| Medical Sciences | 8.3 | // | 2.2 | // |
| Agricultural and VeterinarySciences | // | // | 2.2 | // |
| Civil Engineering and Architecture | 8.3 | // | 4.3 | // |
| Industrial and Information Engineering | 6.2 | // | 6.5 | // |
| Antiquity, Philological-Literary, and      Historical-Artistic Sciences | 4.2 | // | // | // |
| Historical, Philosophical, Pedagogical,      and Psychological Sciences | 14.6 | // | 32.6 | // |
| Legal Sciences | 4.2 | // | 4.3 | // |
| Economic and Statistical Sciences | 8.3 | // | 4.3 | // |
| Political and Social Sciences | // | // | // | // |
| Not Applicable | 41.7 | // | 37 | // |
| Total (*N*) | 48 | 62 | 46 | 49 |

**Table 8.** *Study 5*

|  | **Laypeople** | **Chemists** |
| --- | --- | --- |
| Birth Sex |  |  |
| Male | 36.6 | 41.2 |
| Female | 62.0 | 58.8 |
| Intersex | 1.4 | // |
| Gender |  |  |
| Man | 97.7 | 40.4 |
| Woman | 61.2 | 58.8 |
| Queer | 2.2 | 0.7 |
| Trans | 1.1 | // |
| Other | 0.4 | // |
| Total *(N*) | 276 | 277 |

**1. 1. Education levels of participants (laypeople and chemists) across the studies**

*Note.* Tables 9 – 13 report the percentage of participants for each education level in columns, along with the total number of participants for each group (laypeople and chemists) for each study and—where relevant—each specific task.

**Table 9.** *Experiment 1 Part 1*

|  | **Water** | **Non-Water** |
| --- | --- | --- |
| None/Primary | // | // |
| Junior High School | // | // |
| High School | 11.8 | 46.7 |
| Bachelor Degree | 64.7 | 46.7 |
| Master Degree | 23.5 | 6.7 |
| PhD or other | // | // |
| Total (*N*) | 17 | 15 |

**Table 10.** *Experiment 1.1A*

|  | **Water** | **Non-Water** |
| --- | --- | --- |
| None/Primary | // | // |
| Junior High School | // | 6.7 |
| High School | 26.7 | 26.7 |
| Bachelor Degree | 66.7 | 53.3 |
| Master Degree | 6.7 | 13.3 |
| PhD or other | // | // |
| Total (*N*) | 15 | 15 |

**Table 11.** *Study 1 Part 2*

|  | **Water** | | **Non-water** | |
| --- | --- | --- | --- | --- |
|  | **Laypeople** | **Chemists** | **Laypeople** | **Chemists** |
| None/Primary | // | // | // | // |
| Middle School | 26.1 | // | 13.6 | // |
| High School | 34.8 | // | 31.8 | // |
| Bachelor Degree | 30.4 | // | // | // |
| Master Degree | 8.7 | 30.4 | 22.7 | 43.5 |
| PhD or other | // | 69.6 | // | 56.5 |
| Total (*N*) | 23 | 23 | 22 | 23 |

*Note*. A chi-squared test showed that chemists and non-chemists significantly differed in terms of education both in the water, $\chi^{2}$(4) = 39.778, *p* < .0001, and in the non-water task, $\chi^{2}$(4) = 31.66, *p* < .0001. Specifically, post-hoc tests adjusted with Bonferroni’s correction showed that in both experiments there were more chemists with a PhD than non-chemists (water: *p* < .0001; non-water: *p* < .001), and, conversely, more non-chemists with a bachelor’s degree (water: *p* = .041; non-water: *p* = .032) and a high school diploma (water: *p* = .018; non-water: *p* = .032) than chemists. Instead, no differences were found between chemists and non-chemists in the number of participants with a master’s degree (water: *p* = .631; non-water: *p* = 1.00) and with a middle school diploma (water: *p* = .086; non-water: *p* = .667) for both experiments.

**Table 12.** *Study 2*

|  | **Typicality** | | **Centrality** | | **Frequency** | |
| --- | --- | --- | --- | --- | --- | --- |
|  | **Laypeople** | **Chemists** | **Laypeople** | **Chemists** | **Laypeople** | **Chemists** |
| None/Primary | // | // | // | // | // | // |
| Junior High School | 3.8 | // | // | // | 10 | // |
| High School | 30.8 | // | 35 | // | 50 | // |
| Bachelor Degree | 50 | // | 40 | 5 | 25 | // |
| Master Degree | 11.5 | 19.2 | 20 | 20 | 10 | 10 |
| PhD or other | 3.8 | 80.8 | 5 | 75 | 5 | 90 |
| Total (*N*) | 26 | 26 | 20 | 20 | 20 | 20 |

*Note.* A chi-squared test showed that chemists and non-chemists significantly differed in terms of education both for typicality, $\chi^{2}$(4) = 40.682, *p* < .0001, centrality, $\chi^{2}$(3) = 24.694, *p* < .0001, and frequency ratings, $\chi^{2}$(4) = 32.211, *p* < .0001. Specifically, post-hoc tests adjusted with Bonferroni’s correction showed that in all experiments there were more chemists with a PhD than non-chemists (typicality: *p* < .0001; centrality: *p* < .0001; frequency: *p* < .0001), and, conversely, more non-chemists with a high school diploma than chemists (typicality: *p* = .021; centrality: *p* = .028; frequency: *p* = .002). Only for typicality ratings there were more non-chemists with a bachelor’s degree than chemists, *p* < .001. Instead, in all experiments, no differences were found between chemists and non-chemists in the number of participants with a master’s degree (typicality: *p* = 1.00; centrality: *p* = 1.00; frequency: *p* = 1.00), a bachelor’s degree (centrality: *p* = .064; frequency: *p* = .168), and a middle school diploma (typicality: *p* = 1.000; frequency: *p* = 1.00).

**Table 13.** *Study 3*

|  | **Laypeople** | **Chemists** |
| --- | --- | --- |
| None/Primary | // | // |
| Junior High School | // | // |
| High School | 35 | // |
| Bachelor Degree | 25 | // |
| Master Degree | 30 | 20 |
| PhD or other | 10 | 80 |
| Total (*N*) | 20 | 20 |

*Note.* A chi-squared test showed that chemists and non-chemists significantly differed in terms of education, $\chi^{2}$(3) = 23.289, *p* < .0001. Specifically, post-hoc tests adjusted with Bonferroni’s correction showed that there were more chemists with a PhD than non-chemists, *p* < .0001, and, conversely, more non-chemists with a high school diploma then chemists, *p* = .028. Instead, no differences were found between chemists and non-chemists in the number of participants with a master’s degree, *p* = 1.00, and a bachelor’s degree, *p* = .134.

**Table 14.** *Study 4*

|  | **Only partly** | | **Mostly but not entirely** | |
| --- | --- | --- | --- | --- |
|  | **Laypeople** | **Chemists** | **Laypeople** | **Chemists** |
| None/Primary | // | // | // | // |
| Junior High School | // | // | // | // |
| High School | 29.2 | // | 30.4 | // |
| Bachelor Degree | 31.2 | 6.5 | 26.1 | 8.2 |
| Master Degree | 33.3 | 21 | 37 | 34.7 |
| PhD or other | 6.2 | 72.6 | 6.5 | 57.1 |
| Total (*N*) | 48 | 62 | 46 | 49 |

*Note.* A chi-squared test showed that chemists and non-chemists significantly differed in terms of education both for the “only partly” experiment, $\chi^{2}$(3) = 56.563, *p* < .0001, and for the “mostly but not entirely” experiment, $\chi^{2}$(3) = 38.105, *p* < .0001. Specifically, post-hoc tests adjusted with Bonferroni’s correction showed that in both experiments there were more chemists with a PhD than non-chemists (only partly: *p* < .0001; mostly but not entirely: *p* < .0001), and, conversely, more non-chemists with a high school diploma then chemists (only partly: *p* < .0001; mostly but not entirely: *p* < .001). In addition, only for the “only partly” experiment, there were more non-chemists with a bachelor’s degree than chemists, *p* = .005. Instead, no differences were found in both experiments between chemists and non-chemists in the number of participants with a master’s degree (only partly: *p* = 1.00; mostly but not entirely: *p* = 1.00), and only for the “mostly but not entirely” experiment, in the number of chemists and non-chemists with a bachelor’s degree, *p* = .157.

**Table 15.** *Study 5*

|  | **Laypeople** | **Chemists** |
| --- | --- | --- |
| None/Primary | // | // |
| Junior High School | 4.3 | // |
| High School | 29.7 | 0.4 |
| Bachelor Degree | 30.8 | 3.6 |
| Master Degree | 27.2 | 23.1 |
| PhD or other | 8 | 72.9 |
| Total (*N*) | 276 | 277 |

*Note.* A chi-squared test showed that chemists and non-chemists significantly differed in terms of education, $\chi^{2}$(4) = 295.77, *p* < .0001. Specifically, post-hoc tests adjusted with Bonferroni’s correction showed that there were more chemists with a PhD than non-chemists, *p* < .0001, and conversely, more non-chemists with a bachelor’s degree, *p* < .0001, a high school diploma, *p* < .0001, and a middle school diploma, *p* = .004, than chemists. Instead, no differences were found between chemists and non-chemists in the number of participants with a master’s degree, *p* = 1.00.

**S2. Chemists’ frequency of interaction with chemicals**

**Table 16.** *Chemists’* *frequency of interaction with chemicals. Responses are given in percentages, along with the total number of participants for each individual study and each task.*

| *How many times a week do you handle or interact with chemical substances or compounds?* | **Study 1.2** | | **Study 2** | **Study 3** | **Study 4** | | **Study 5** |
| --- | --- | --- | --- | --- | --- | --- | --- |
|  | **Water** | **Non-water** |  |  | **Only partly** | **Mostly but not entirely** |  |
| Never (less than once a week) | // | // | // | // | 1.6 | 2 | 0.7 |
| Rarely (between once and twice a week | 3.4 | // | 7.6 | 10 | 8.1 | 8.2 | 7.6 |
| Often (from two to three times a week) | 8.7 | 21.7 | 4.5 | 10 | 11.3 | 28.6 | 13.7 |
| Very often (almost everyday) | 13 | 30 | 18.2 | 30 | 21 | 20.4 | 20.9 |
| Always (everyday) | 73 | 47.8 | 69.7 | 50 | 58.1 | 40.8 | 57 |
| Total (*N*) | 23 | 23 | 66 | 20 | 62 | 49 | 277 |

**S3. Chemophobia and Knowledge of Toxicological principles questionnaires**

The short version of the Chemophobia questionnaire developed by Bearth, Kwon & Siegrist (2021) consists of six items comprising statements targeting irrational fear of chemicals and related health concerns. Participants are asked to rate their agreement with each statement on a scale from 1 = “completely disagree” to 6 = “completely disagree”, such that higher scores reflect higher levels of chemophobia. Items that were not translated into Italian in previous versions of the questionnaire were back-translated from two authors (CM and MA, respectively Italian and English native speakers fluently speaking both languages). Table 1 reports each statement along with its Italian translation.

**Table 17.** *Italian adaptation of Chemophobia questionnaire (Bearth et al., 2021).*

| **English statement** | **Italian translation** |
| --- | --- |
| I would like all chemical substances to be risk-free. | *Mi piacerebbe che tutte le sostanze chimiche fossero prive di rischi.* |
| I believe that chemical substances are the main reason why people suffer from cancer. | *Credo che le sostanze chimiche siano il motivo principale per cui le persone soffrono di cancro.* |
| Chemical substances scare me. | *Le sostanze chimiche mi spaventano.* |
| I do everything I can to avoid in my daily life contact with chemical substances. | *Faccio qualsiasi cosa per ridurre il contatto con le sostanze chimiche nella mia vita quotidiana.* |
| I would like to live in a world where chemical substances don’t exist. | *Vorrei vivere in un mondo in cui non esistono sostanze chimiche.* |
| In a world without chemical substances, there would be no environmental disasters. | *In un mondo senza sostanze chimiche, non ci sarebbero disastri ambientali.* |

The questionnaire assessing people’s “Knowledge of toxicological principles” (Bearth, Saleh & Siegrist, 2019) is composed of seven incorrect and five correct statements for which participants are asked to indicate, to the best of their knowledge, whether the statements they are presented with are “true”, “false”, or whether they “do not know”. The questionnaire is validated also in Italian. Table 8 reports each statement along with its Italian translation, and correct answers.

**Table 18.** *Knowledge of toxicological principles questionnaire (Bearth et al., 2019), its Italian translation, and correct answers.*

| **English statement** | **Italian translation** | **Correct answer** |
| --- | --- | --- |
| The dangerousness of a chemical substance does not only depend on the amount that you are exposed to, but also on the frequency with which you are exposed to this chemical substance. | *La tossicità di una sostanza dipende non solo dalla quantità ma anche dalla frequenza con cui si è esposti/e alla sostanza.* | true |
| Both synthetic and natural chemical substances can cause cancer in humans. | *Possono provocare il cancro alle persone sia le sostanze sintetiche che quelle naturali.* | true |
| The human body can deal with the toxicity of natural chemical substances but not with that of synthetic chemical substances. | *Il corpo umano può gestire la tossicità di sostanze chimiche naturali ma non quella di sostanze chimiche sintetiche.* | false |
| Solely consumer products with synthetic chemical substances are labelled with danger symbols. | *Solo prodotti per uso domestico che sono stati prodotti in modo sintetico presentano simboli di pericolo.* | false |
| The dose at which a toxic synthetic chemical substance causes illness is always smaller than that of a toxic natural chemical substance. | *La dose con cui una sostanza sintetica velenosa causa una malattia è sempre più bassa di quella di una sostanza naturale velenosa.* | false |
| The chemical structure of the synthetically produced salt (NaCl) is exactly the same as that of salt found naturally in the sea. | *La struttura chimica del sale che è stato prodotto in modo sintetico (NaCl) è la stessa del sale marino.* | true |
| Being exposed to a toxic synthetic chemical substance is always dangerous, no matter what the level of exposure is. | *Indipendentemente dalla quantità è sempre pericoloso essere esposti/e ad una sostanza chimica sintetica velenosa.* | false |
| Synthetic chemical substances accumulate in the human body to a greater extent than natural chemical substances. | *Le sostanze sintetiche si accumulano maggiormente nel corpo umano rispetto a quelle naturali.* | false |
| Synthetic chemical substances from consumer products are the main cause of allergies in humans. | *Le sostanze sintetiche in prodotti di consumo sono la causa principale delle allergie.* | false |
| A small amount of a toxic chemical substance in a consumer product is not necessarily harmful. | *Una piccola quantità di una sostanza velenosa in un prodotto di consumo non è necessariamente nociva.* | true |
| When a substance causes cancer in animals, then it definitely causes cancer in humans. | *Se una sostanza provoca il cancro agli animali lo provoca in ogni caso anche alle persone.* | false |
| Any chemical substance – synthetic or natural – can cause death if a person is exposed to it in large amounts. | *Ogni sostanza – sia sintetica che naturale – in grandi quantità può portare alla morte.* | true |

**S4. Experiment 2: Correlation matrix**

**Table 19.** *Means, standard deviations, and correlations with confidence intervals between variables (i.e., Typicality, Centrality, Frequency, and H*_2_*O Estimates) both within and between experimental groups (Laypeople, Chemists) for Experiment 2.*

| Variable | *M* | *SD* | 1 | 2 | 3 | 4 | 5 | 6 | 7 |
| --- | --- | --- | --- | --- | --- | --- | --- | --- | --- |
|  |  |  |  |  |  |  |  |  |  |
| 1. Laypeople Typicality | 4.87 | 0.90 |  |  |  |  |  |  |  |
|  |  |  |  |  |  |  |  |  |  |
| 2. Laypeople Centrality | 3.81 | 1.41 | .87** |  |  |  |  |  |  |
|  |  |  | [.78, .92] |  |  |  |  |  |  |
|  |  |  |  |  |  |  |  |  |  |
| 3. Laypeople Frequency | 4.27 | 0.89 | .92** | .79** |  |  |  |  |  |
|  |  |  | [.86, .95] | [.65, .88] |  |  |  |  |  |
|  |  |  |  |  |  |  |  |  |  |
| 4. Laypeople H_2_O Estimates | 68.69 | 9.45 | .03 | .03 | -.01 |  |  |  |  |
|  |  |  | [-.31, .25] | [-.25, .32] | [-.41, .14] |  |  |  |  |
|  |  |  |  |  |  |  |  |  |  |
| 5. Chemists Typicality | 4.87 | 0.90 | .83** | .85** | .76** | .14 |  |  |  |
|  |  |  | [.71, .90] | [.75, .91] | [.61, .86] | [-.15, .41] |  |  |  |
|  |  |  |  |  |  |  |  |  |  |
| 6. Chemists Centrality | 3.77 | 1.37 | .73** | .88** | .66** | .17 | .80** |  |  |
|  |  |  | [.56, .84] | [.80, .93] | [.46, .79] | [-.11, .44] | [.66, .88] |  |  |
|  |  |  |  |  |  |  |  |  |  |
| 7. Chemists Frequency | 4.02 | 1.16 | .64** | .56** | .69** | -.10 | .64** | .60** |  |
|  |  |  | [.43, .78] | [.33, .73] | [.51, .82] | [-.37, .19] | [.44, .78] | [.38, .76] |  |
|  |  |  |  |  |  |  |  |  |  |
| 8. Chemists H_2_O Estimates | 96.08 | 3.24 | .06 | .13 | -.02 | .94** | .24 | .27 | .01 |
|  |  |  | [-.22, .34] | [.15, .40] | [-.30, .26] | [.90, .97] | [-.04, .49] | [-.008, .52] | [-.27, .30] |
|  |  |  |  |  |  |  |  |  |  |

*Note.* *M* and *SD* are used to represent mean and standard deviation, respectively. Values in square brackets indicate the 95% confidence interval for each correlation. ** indicates *p* < .05.

**Table 19a*.*** *Cronbach’s Alphas (α) of Interrater Reliability for Typicality, Centrality and Frequency ratings provided by Laypeople and Chemists of Experiment 2*.

|  | **Laypeople** |  | **Chemists** |
| --- | --- | --- | --- |
|  | *α* |  | *α* |
| Typicality | 0.95 |  | 0.98 |
| Centrality | 0.91 |  | 0.94 |
| Frequency | 0.95 |  | 0.89 |

**S5. Experiment 3: Multidimensional Scaling Solutions for laypeople and chemists similarity ratings**

**S6. Experiment 4**

**“X is a type of water” vs “X is only partly water”**

**Model 1: “X is a type of water”.** We found a significant interaction between Group and Liquid Type, $\chi^{2}$(1) = 10.08, *p* = .001. Post-hoc comparisons showed chemists are less likely to judge non-water examples as “types of water” than non-chemists, *z* = -2.289, SE = 0.544, *p* = .022, while there was no difference in judgements on water examples, *p* = .591

**Model 2: “X is only partly water”.** We found a significant interaction between Group and Liquid Type, $\chi^{2}$(1) = 10.23, *p* = .001. Post-hoc comparisons showed chemists are more likely to judge non-water examples as “only partly water” than non-chemists, *z* = -4.136, SE = 0.544, *p* < .001. On average, chemists are also slightly more likely than non-chemists to judge water examples as being “only partly water”, but this did not reach significance, *p* = .053.

**“X is a type of water” vs “X is mostly but not entirely water”**

**Model 1: “X is a type of water”.** We found a significant main effect of Liquid Type, $\chi^{2}$(1) = 9.97, *p* =.001, showing that overall non-water examples were less likely to be judged as types of water than water examples, EMM non-water = -1,99; SE = .43; EMM water = 2.25; SE = 1.38. No other significant main effects or interaction emerged, all *p*_s_ > .287.

**Model 2: “X is mostly but not entirely water”.** There was a significant main effect of Group, $\chi^{2}$ (1) = 10.10, *p* = .001, showing that overall chemists were slightly more likely to give “mostly but not entirely” judgements than non-chemists**,** EMM chemists = .27; SE = .776; EMM non chemists = .26; SE = .780. No other significant main effect or interaction emerged, all *p*_s_ > .251.

**S7. Chemophobia and Knowledge of Toxicological Principles across laypeople and chemists**

While chemicals constitute an essential part of our everyday lives, they might be perceived as dangerous, and as something that should be avoided at all costs. This irrational tendency has been named chemophobia (review in Rollini, Falciola & Tortorella, 2022; Saleh, Bearth & Siegrist, 2019; Siegrist & Bearth, 2019), and has been found to be associated with limited knowledge about toxicological principles (Bearth, Saleh & Siegrist, 2019). This seems to be especially relevant in Italy, as Bearth, Saleh, and Siegrist (2019) found that Italian participants displayed—together with French—the highest chemophobia levels across eight European countries. While this trend might be worrying, there is also evidence that experts tend to overestimate the degree of chemophobia among the general public (Royal Society of Chemistry, 2015), further highlighting the discrepancies in perceptions and misconceptions underlying this phenomenon. Finally, it has been proposed that the language chemists use to talk about molecules—which is mainly composed of formulas and structures—might contribute to endow non-specialists with feelings of aversion (Serban et al., 2018; Royal Society of Chemistry, 2015; Francl, 2013).

Here we tested Chemophobia and Knowledge of Toxicological principles across Italian laypeople and chemists using previously employed questionnaires (Bearth, Kwon & Siegrist, 2021; Bearth, Saleh & Siegrist, 2019). Since we did not have specific hypotheses on the implications of chemophobia scores or knowledge of toxicological principles for our research questions, we will simply report the results for illustrative purposes. Future work might deepen aspects related to the link between these two constructs and essentialist perspectives of natural kinds. All the participants enrolled in the studies filled out the Chemophobia and Knowledge of Toxicological principles questionnaires (see ¶1).

*7.1. Data analysis*

Responses to the Chemophobia questionnaire were analysed using a mixed ordinal regression model. The model featured Chemophobia scores as dependent variable, Group as categorical predictor, and random intercepts for participants.

Responses to the Knowledge of chemicals questionnaire were analysed using a mixed binomial regression. The model featured only correct vs incorrect responses as dependent variable, Group as categorical predictor, and random intercepts for participants.

*7.2. Results*

*Chemophobia.* On average, laypeople were more chemophobic than chemists, *M* laypeople = 3.27; *SD* = 1.77; *M* chemists = 1.86; *SD* = 1.57. The model showed a significant main effect of Group, $\chi^{2}$(1) = 259.81, *p* <.001, confirming this trend, *b* = 2.084, SE = .120, *z* = 17.27, *p* <.0001.

*Knowledge.* Overall, laypeople gave 49.96% of correct responses, 20.95% of incorrect responses, and were unsure about the 29.07% of questions. Chemists, on the other hand, gave 85.28% of correct responses, 8.21% of incorrect responses, and were unsure about 6.49% of questions. The model showed a significant main effect of Group, $\chi^{2}$(1) = 269.42, *p* <. 0001, showing that laypeople gave more incorrect answers compared to chemists, *b* = -1.529, SE = .093, *z* = -16.41, *p* <. 0001.

While these findings might not be surprising, it is interesting to note that compared to the average Italian chemophobia score reported in Bearth et al. (2019) (*M* = 4.30; *SD* = 1.14), we observed a lower rate of chemophobia among our sample of non-chemists, although there are consistent differences in the sample sizes of the two studies.

**References**

BMRB, T. (2015). *Public attitudes to chemistry*. Research Report.

Francl, M. (2013). How to counteract chemophobia. *Nature Chemistry*, *5*(6), 439-440.

Serban, B. C., Buiu, O., Bumbac, M., Nicolescu, C. M., & Cobianu, C. (2018). Chemistry-the Journey from Central Science to Chemofobia; How Should We Address This?. *Journal of Science & Arts*, *18*(4).

Rollini, R., Falciola, L., & Tortorella, S. (2022). Chemophobia: A systematic review. *Tetrahedron*, *113*, 132758.

**Appendix**

**Data Analysis**

*1. Experiment 1 Part 1*

Free-listing data were preprocessed as follows: all capital letters were converted into small caps, the spelling was standardised, and clear synonyms were collapsed (e.g., “acqua gassata” and “acqua frizzante”, both Italian synonyms of *sparkling water*).

*2.Experiment 1 Part 2*

Data processing was carried out using ‘tidyverse’ (Wickham et al., 2019), ‘dplyr’ (Wickham, François and Müller, 2022), and ‘widyr’ (Robinson & Silge, 2022) R packages. Data visualisation was implemented using ‘ggplot2’ (Wickham, 2016) and ‘ggpubr’ (Kassambara, 2020), and ‘ggrepel’ (Slowikowski, 2021) R packages.

To assess our main hypothesis we fitted one linear mixed model, implemented through ‘lme4’ (Bates et al., 2015) R package. The model featured Estimated Percentages as dependent variable, Group (chemists vs non-chemists), Type of Liquid (water vs non-water) and their interaction as independent variables, and participants and liquids as random intercepts. Main effects and their interaction were assessed with Type II Wald chisquare tests implemented through the Anova() function of the R’s package ‘car’ (Fox & Weisberg, 2019). Finally, to assess whether chemists’ estimates aligned more than non-chemists estimates with the actual H_2_O percentages of each liquid, we used Pearson’s correlations.

*3.Experiment 2*

Data were analysed using Pearson’s correlations and linear regressions. To assess the percentage of variance explained by each dependent variable, Multiple *R*^2^ was computed. For each group, we calculated correlations between typicality ratings and estimates of H_2_O provided by an independent sample of participants (i.e., laypeople and chemists of Experiment 1 Part 2, henceforth H_2_O estimates), correlations between centrality ratings and H_2_O estimates, correlations between frequency ratings and H_2_O estimates, and correlations between typicality ratings and centrality ratings. Finally, to test which conceptual properties best explain what each group considers to be typical for the category of water, we fitted separate models with typicality as a dependent variable, iteratively adding each predictor (i.e., centrality, frequency, H_2_O estimates) and calculating the proportion of variance explained by each model.

*4.Experiment 3*

Similarity ratings of pairs of water examples of the two groups (laypeople vs chemists) were first correlated using Pearson’s correlations. Then, to explore possible group differences, they were analysed using a mixed ordinal regression model implemented through the ‘ordinal’ R’s package (Christensen, 2022), and significance of the main effect was assessed with the Anova.clmm() function of the ‘RVAideMemoire’ R’s package (Hervé, 2022). The model featured similarity scores as dependent variable, Group as categorical predictor, and random intercepts for participants and item pairs. For the main analyses, ratings were then averaged across participants within each group and then transformed into a dissimilarity matrix based on Euclidean distances. We sought to probe whether there might be more subtle and qualitative differences in the conceptual structuring of the two groups using Non-metric Multidimensional Scaling (MDS). MDS for each group was performed with the ‘smacof’ R’s package (De Leeuw & Mair, 2009) assuming the Euclidean distance function and the stress-1 loss function (see also Verheyen et al., 2021). However, stress -1 values retrieved from the 2-dimensional MDS solution of both laypeople and chemists ratings were poor (0. 23 for laypeople and 0.24 for chemists, Kruskal & Wish, 1978), so we decided to use Hierarchical Cluster Analysis for better interpreting the data (for the results of the MDS please see SM, section 5). In the paper then we discuss cluster analysis results.

Hierarchical Cluster Analyses (HCA) were performed with the hclust() R base function using Ward’s clustering method (Murtagh & Legendre, 2014), and Hopkins’ clusterability Indexes (Lawson & Jurs, 1990) and data visualisation were carried out with the ‘factoextra’ R package (Kassambara & Mundt, 2020). Clusters were identified upon visual inspection.

*5.Experiment 4 Part 1 and 2*

Sentence acceptability judgements were analysed with binomial generalised mixed models with acceptability judgements (yes vs no) as response variable, Group (chemists vs non-chemists), Liquid Type (water vs non-water), Task (Task 1: “X is a type of water”; Task 2: “X is only partly water”), and their interaction as categorical predictors, and random intercepts for participants and items^[[1]](#footnote-1)^. Data were modelled using the glmer() function of the ‘lme4’ R package (Bates, Mächler, Bolker & Walker, 2015). Significance of main effects was assessed using the Anova() function of the ‘car’ R package with type II Wald Chisquare tests, and post-hoc comparisons were carried out using the ‘emmeans’R package (Lenth, 2021) with Tukey’s adjustments for multiple comparisons.

*6. Experiment 5*

Ratings were analysed using a mixed ordinal regression model implemented through the ‘ordinal’ R’s package (Christensen, 2022), and significance of the main effects was assessed with the Anova.clmm() function of the ‘RVAideMemoire’ R’s package (Hervé, 2022). The model featured Abstractness scores as dependent variable, Group, Concept (H_2_O vs water), and their interaction as categorical predictors, and random intercepts for participants. Post-hoc comparisons were performed with ‘emmeans’ R’s package (Lenth, 2021).

**References**

Bates, D., Mächler, M., Bolker, B. & Walker, S. (2015). “Fitting Linear Mixed-Effects Models Using lme4.” Journal of Statistical Software, 67(1), 1–48. doi:10.18637/jss.v067.i01.

Christensen, R. H. B. (2022). ordinal - Regression Models for Ordinal Data. R package version 2022.11-16. <https://CRAN.R-project.org/package=ordinal>.

De Leeuw, J. &  Mair, P. (2009). Multidimensional Scaling Using Majorization: SMACOF in R. Journal of Statistical Software, 31(3), 1-30. URL <https://www.jstatsoft.org/v31/i03/>.

Fox, J. & Weisberg, S. (2019). An {R} Companion to Applied Regression, Third Edition. Thousand Oaks. CA: Sage. URL: <https://socialsciences.mcmaster.ca/jfox/Books/Companion/>

Hervé, M. (2022). RVAideMemoire: Testing and Plotting Procedures for Biostatistics. R package version 0.9-81-2. <https://CRAN.R-project.org/package=RVAideMemoire>

Kassambara, A. (2020). ggpubr: 'ggplot2' Based Publication Ready Plots. R package version 0.4.0. <https://CRAN.R-project.org/package=ggpubr>

Kassambara, A. & Mundt, F. (2020). factoextra: Extract and Visualize the Results of Multivariate Data Analyses. R package version 1.0.7. https://CRAN.R-project.org/package=factoextra

Kruskal, J. and Wish, M. (1978). Multidimensional scaling. Beverly Hills, Calif.: Sage Publications.

Lakens, D. (2024). When and how to deviate from a preregistration. *Collabra: Psychology*, *10*(1), 117094.

Lawson, R. G., & Jurs, P. C. (1990). New index for clustering tendency and its application to chemical problems. *Journal of chemical information and computer sciences*, *30*(1), 36-41.

Lenth, R. V. (2021). emmeans: Estimated Marginal Means, aka Least-Squares Means. R package version 1.7.1-1. <https://CRAN.R-project.org/package=emmeans>

Murtagh, F., & Legendre, P. (2014). Ward’s hierarchical agglomerative clustering method: which algorithms implement Ward’s criterion?. *Journal of classification*, *31*, 274-295.

R Core Team (2019) R: A Language and Environment for Statistical Computing. R Foundation for Statistical Computing, Vienna, Austria.
https://www.R-project.org/

Robinson, D & Silge, J. (2022). widyr: Widen, Process, then Re-Tidy Data. R package version 0.1.5. <https://CRAN.R-project.org/package=widyr>

Slowikowski, K. (2021). ggrepel: Automatically Position Non-Overlapping Text Labels with 'ggplot2'. R package version 0.9.1. <https://CRAN.R-project.org/package=ggrepel>

Wickham, H. (2016). ggplot2: Elegant Graphics for Data Analysis. Springer-Verlag New York.

Wickham, H., Averick, M., Bryan, J., Chang, W., McGowan, L.D., François, R., …, & Yutani, H. (2019). “Welcome to the tidyverse.” _Journal of Open Source Software_,*4*(43), 1686. doi: 10.21105/joss.01686 (URL: <https://doi.org/10.21105/joss.01686>).

Wickham, H., Romain François, R., Henry, L. & Müller, K. (2022). dplyr: A Grammar of Data Manipulation. R package version 1.0.8. <https://CRAN.R-project.org/package=dplyr>

**Table 1**. *Complete list of Italian water and non-water examples and their English translations.*

| **Italian water example** | **English Translation** | **Italian non-water example** | **English Translation** |
| --- | --- | --- | --- |
| *acqua di sorgente* | babbling brook water | *lacrime* | tears |
| *acqua effervescente* | fizzy water | *sudore* | sweat |
| *acqua fresca* | fresh water | *tisana* | herbal tea |
| *acqua in bottiglia* | bottled water | *pipi* | pee |
| *acqua minerale* | mineral water | *saliva* | saliva |
| *acqua naturale* | natural water | *the caldo* | hot tea |
| *acqua oligominerale* | oligomineral water | *collirio* | eyedrops |
| *acqua potabile* | potable water | *vino* | wine |
| *acqua di fonte* | spring water | *aranciata* | orange juice |
| *acqua filtrata* | filtered water | *succo di frutta* | fruit juice |
| *acqua frizzante* | sparkling water | *acqua ossigenata* | hydrogen peroxide |
| *acqua in vetro* | glass water | *Sprite* | Sprite |
| *acqua cristallina* | crystalline water | *birra* | beer |
| *acqua di fiume* | river water | *idrogeno liquido* | liquid hydrogen |
| *acqua distillata* | distilled water | *gassosa* | soda |
| *acqua dolce* | sweet water | *coca cola* | coke |
| *acqua marina* | sea water | *chinotto* | chinotto |
| *acqua calcarea* | calcareous water | *caffè* | coffee |
| *acqua di lago* | lake water | *sangue* | blood |
| *acqua oceanica* | ocean water | *salsa di soia* | soy sauce |
| *acqua santa* | saint water | *disinfettante* | disinfectant |
| *acqua termale* | thermal water | *candeggina* | bleach |
| *acqua piovana* | rain water | *vodka* | vodka |
| *acqua salata* | salt water | *balsamo capelli* | hair conditioner |
| *acqua di stagno* | pond water | *acetone* | nail polish remover |
| *acqua sulfurea* | sulfuric water | *linfa* | lymph |
| *acqua torbida* | muddy water | *aceto* | vinegar |
| *acqua di rubinetto* | tap water | *olio extravergine* | extravirgin olive oil |
| *acqua di piscina* | swimming pool water | *lozione struccante* | make-up removing lotion |
| *acqua saponata* | soapy water | *soluzione fisiologica* | saline solution |
| *acqua stagnante* | stagnant water | *soluzione lenti a contatto* | contact lens cleaner |
| *acqua demineralizzata* | demineralized water | *collutorio* | mouthwash |
| *acqua inquinata* | polluted water | *lozione dopobarba* | aftershave lotion |
| *acqua depurata* | purified water | *ricarica fluida accendino* | lighter fluid |
| *acqua acidulata* | acidulated water | *repellente zanzare* | mosquito repellent |
| *acqua di termosifone* | radiator water | *limonata* | lemonade |
| *acqua di fognatura* | sewer water | *brodo di carne* | meat broth |
| *acqua di radiatore* | car radiator water | *latte scremato* | skim milk |
| *acqua di pozzo* | well water | *latte avena* | oat milk |
| *acqua di neve sciolta* | water from melted rain | *spray gola* | throat spray |
| *acqua non purificata* | unpurified water | *sgrassatore cucina* | kitchen spray cleaner |
| *acqua della vasca da bagno* | bath water | *tonico viso* | tonic for face |
| *acqua di cascata* | waterfall water | *gin* | gin |
| *acqua di ruscello* | stream water | *profumo* | perfume |
| *acqua di montagna* | mountain water | *olio essenziale* | essential oil |
| *acqua di pozzanghera* | puddle water | *latte detergente* | face cleanser |
| *acqua di fontana* | fountain water | *limoncello* | limoncello |

**Table 2.** *List of water examples used as stimuli in Experiment 3, and their English translations.*

| **Italian water example** | **English Translation** |
| --- | --- |
| *acqua minerale* | mineral water |
| *acqua naturale* | natural water |
| *acqua di fiume* | river water |
| *acqua distillata* | distilled water |
| *acqua calcarea* | calcareous water |
| *acqua di lago* | lake water |
| *acqua piovana* | rain water |
| *acqua salata* | salt water |
| *acqua di stagno* | pond water |
| *acqua sulfurea* | sulfuric water |
| *acqua torbida* | muddy water |
| *acqua di rubinetto* | tap water |
| *acqua di piscina* | swimming pool water |
| *acqua stagnante* | stagnant water |
| *acqua di termosifone* | radiator water |
| *acqua di fognatura* | sewer water |
| *acqua di radiatore* | car radiator water |
| *acqua di pozzo* | well water |
| *acqua della vasca da bagno* | bath water |
| *acqua di pozzanghera* | puddle water |

1. These data were first analysed separately for each task in keeping with the preregistration plan, as the interaction of interest that was mistakenly omitted in the preregistration plan (please see Lakens, 2024 for deviations from preregistrations). For both Experiment 4 Part 1 and 4 Part 2 we describe results from the full models, as they best target our research question. Results from the other models are reported in the Supplementary Materials (see section 6). [↑](#footnote-ref-1)
